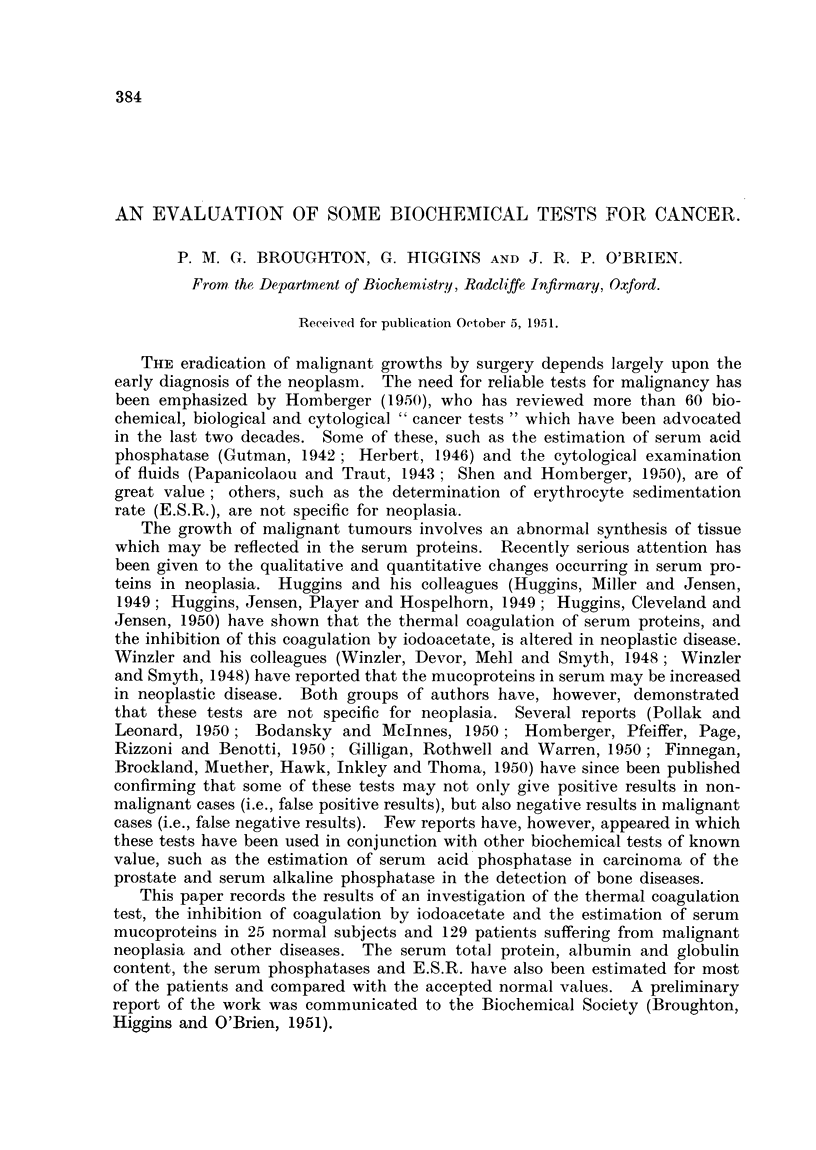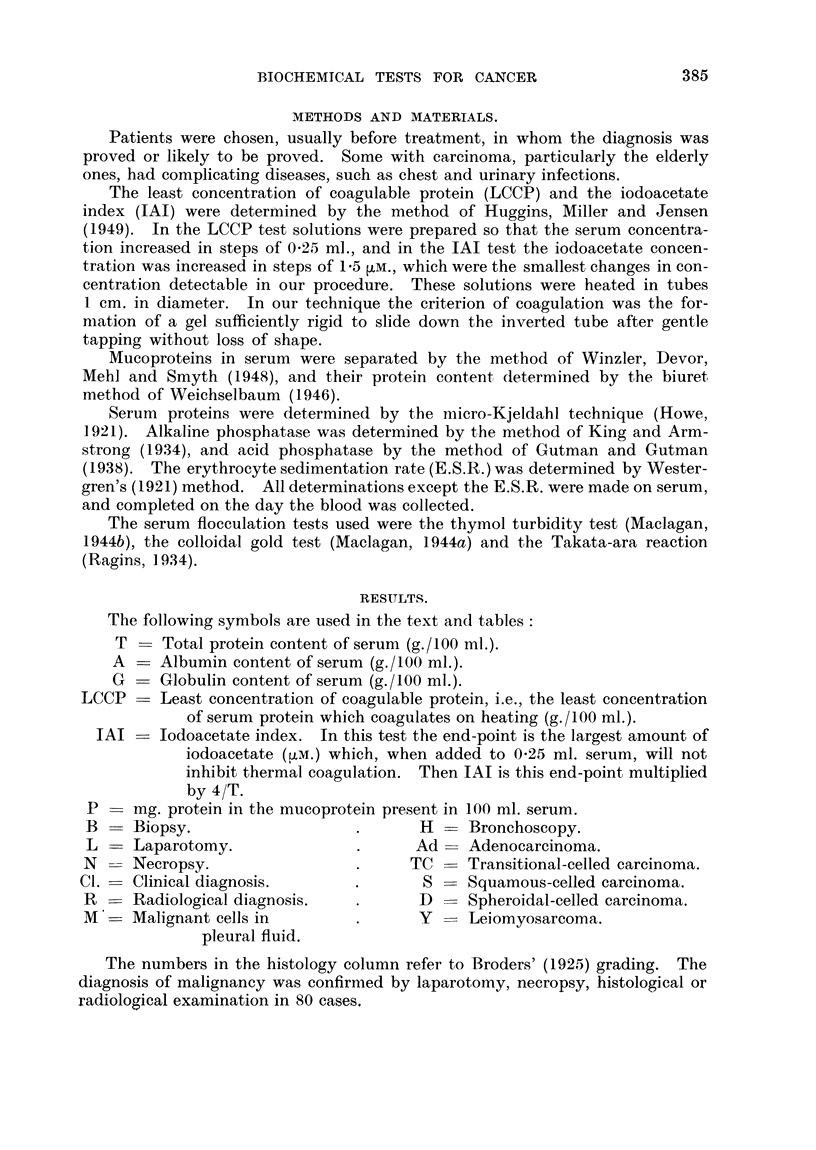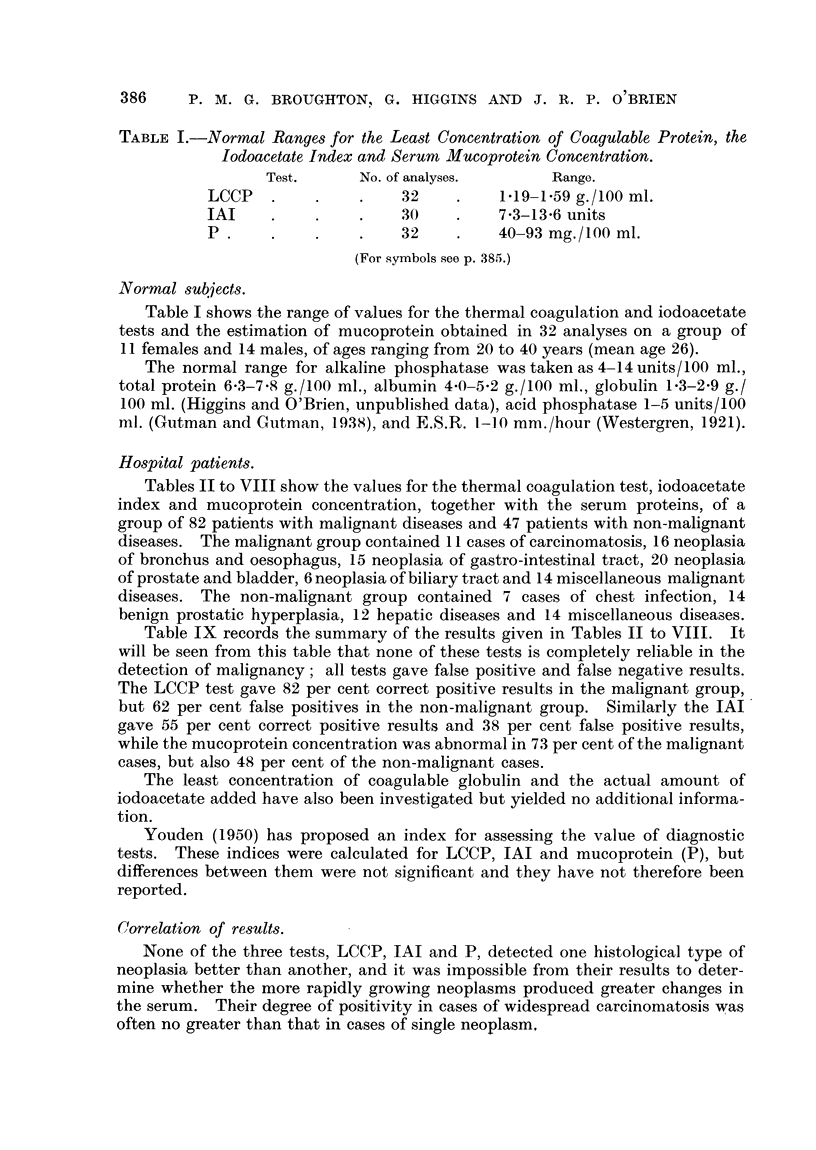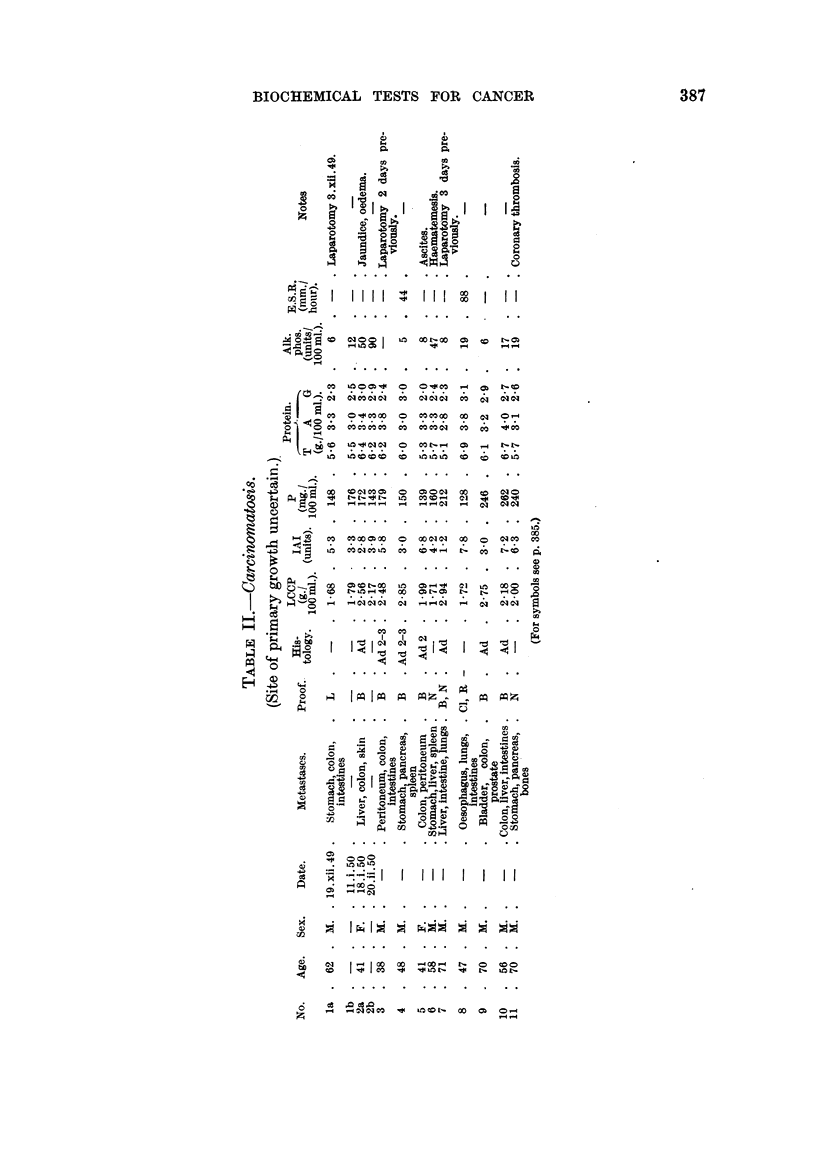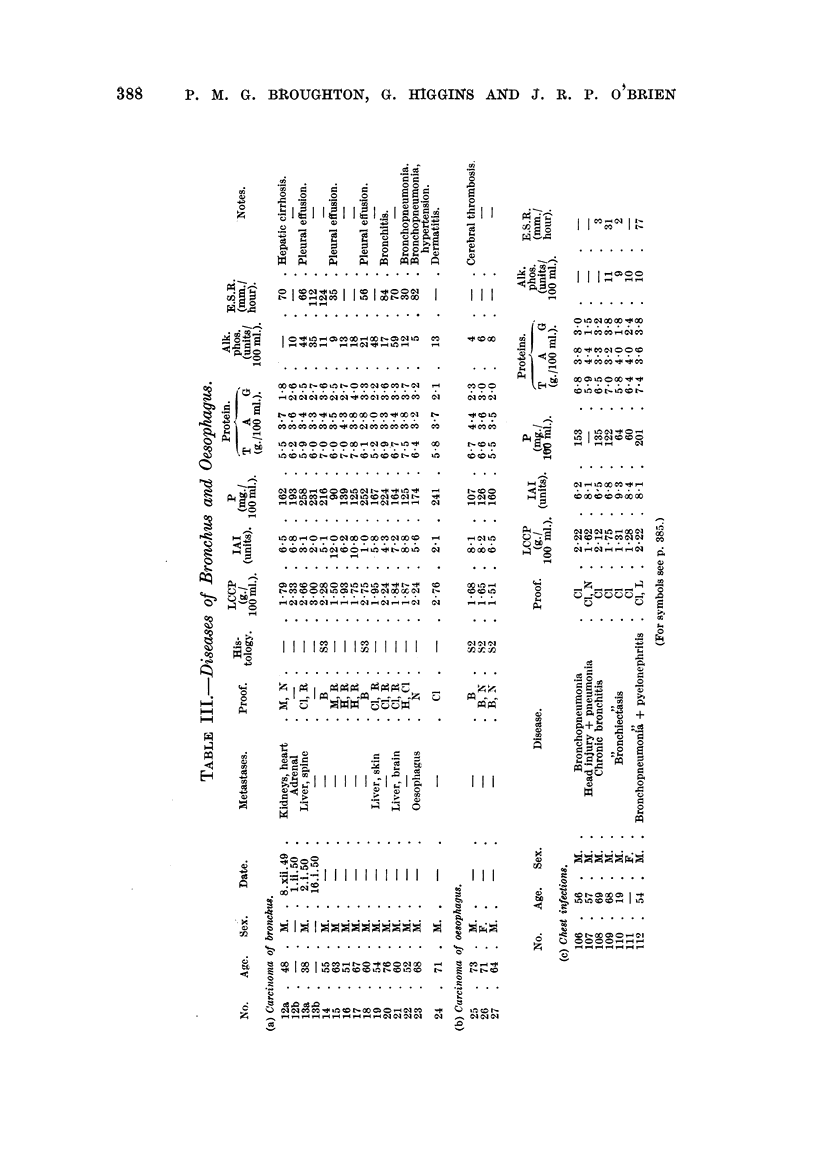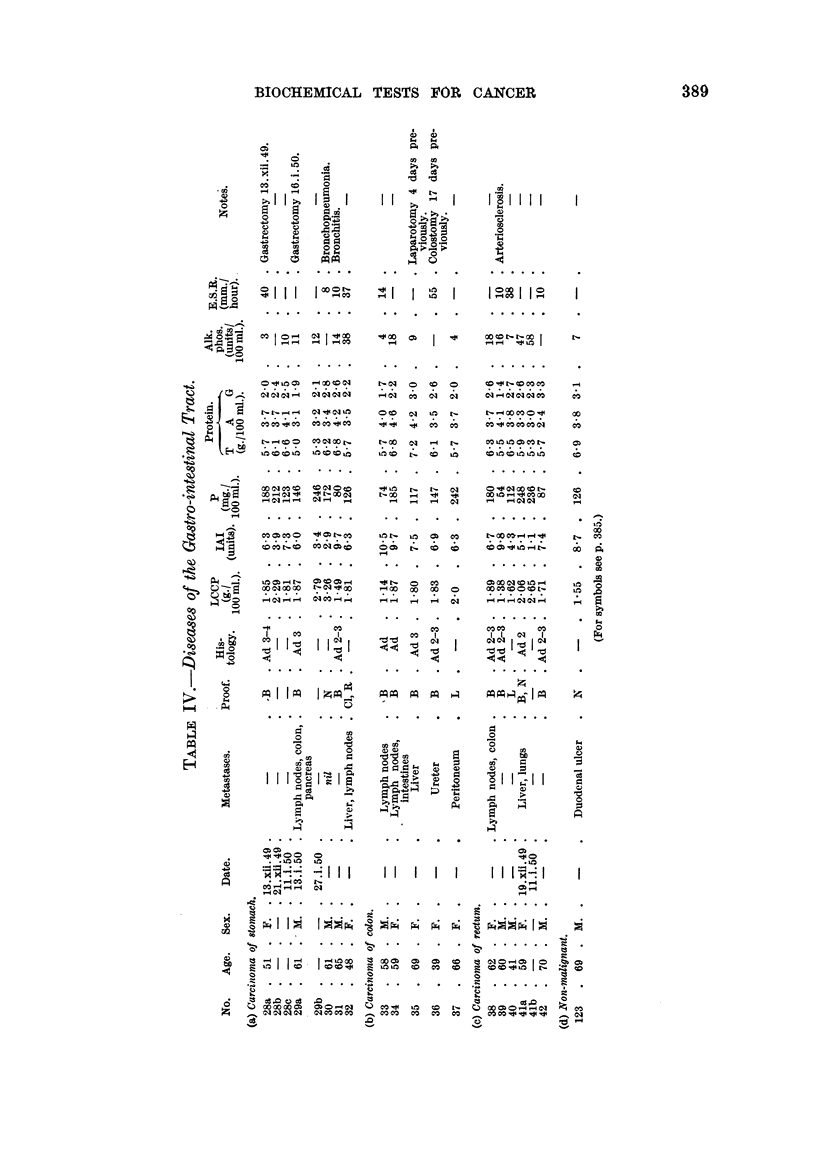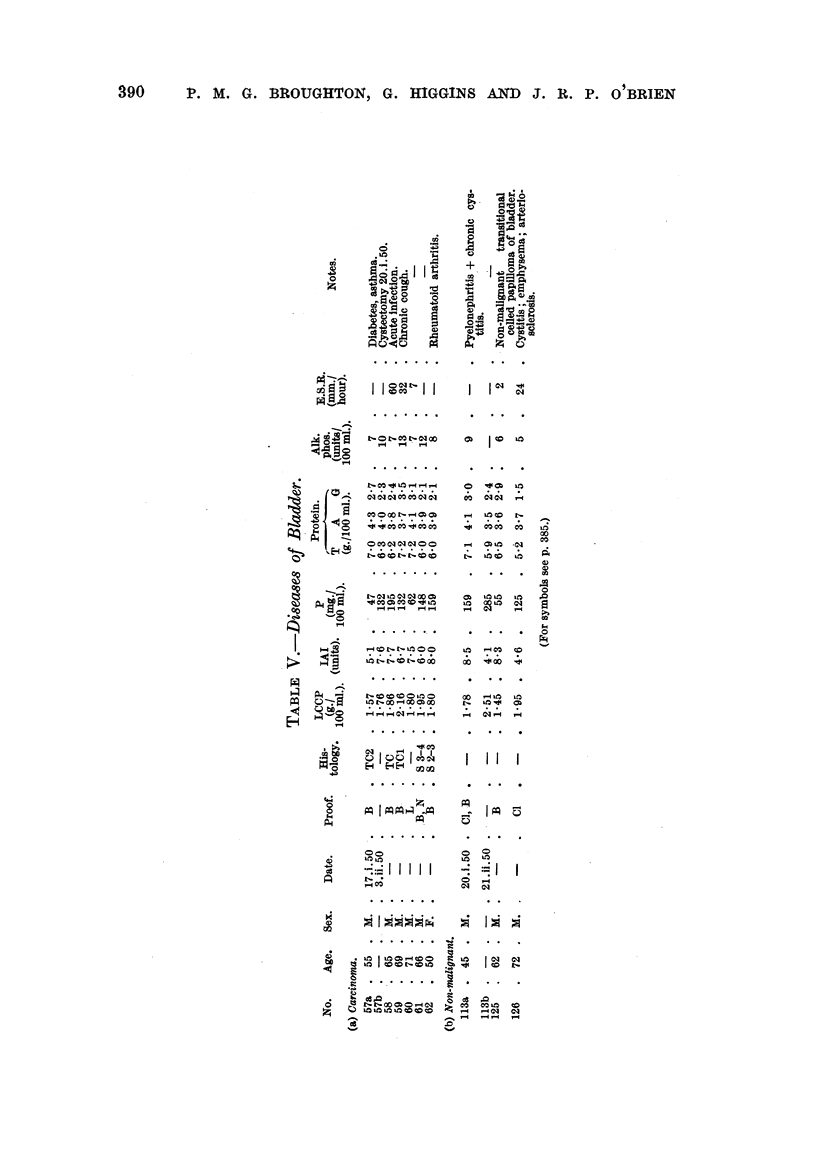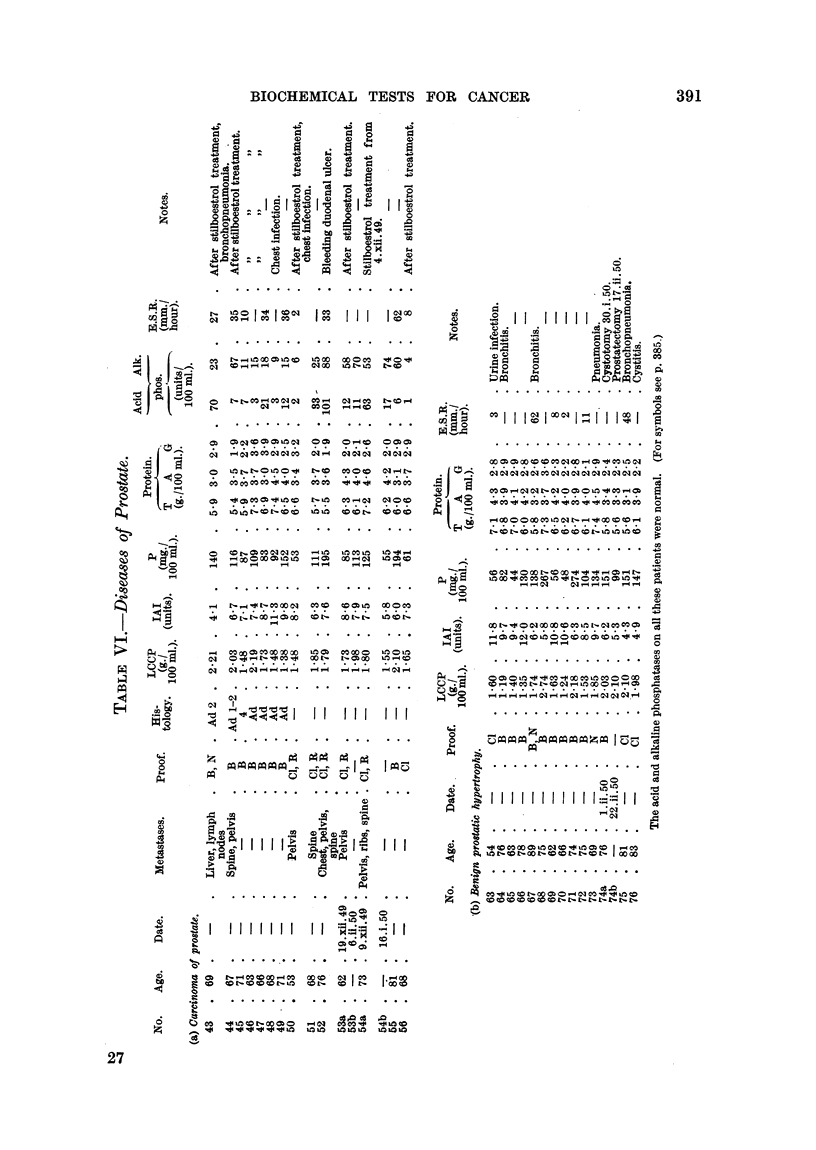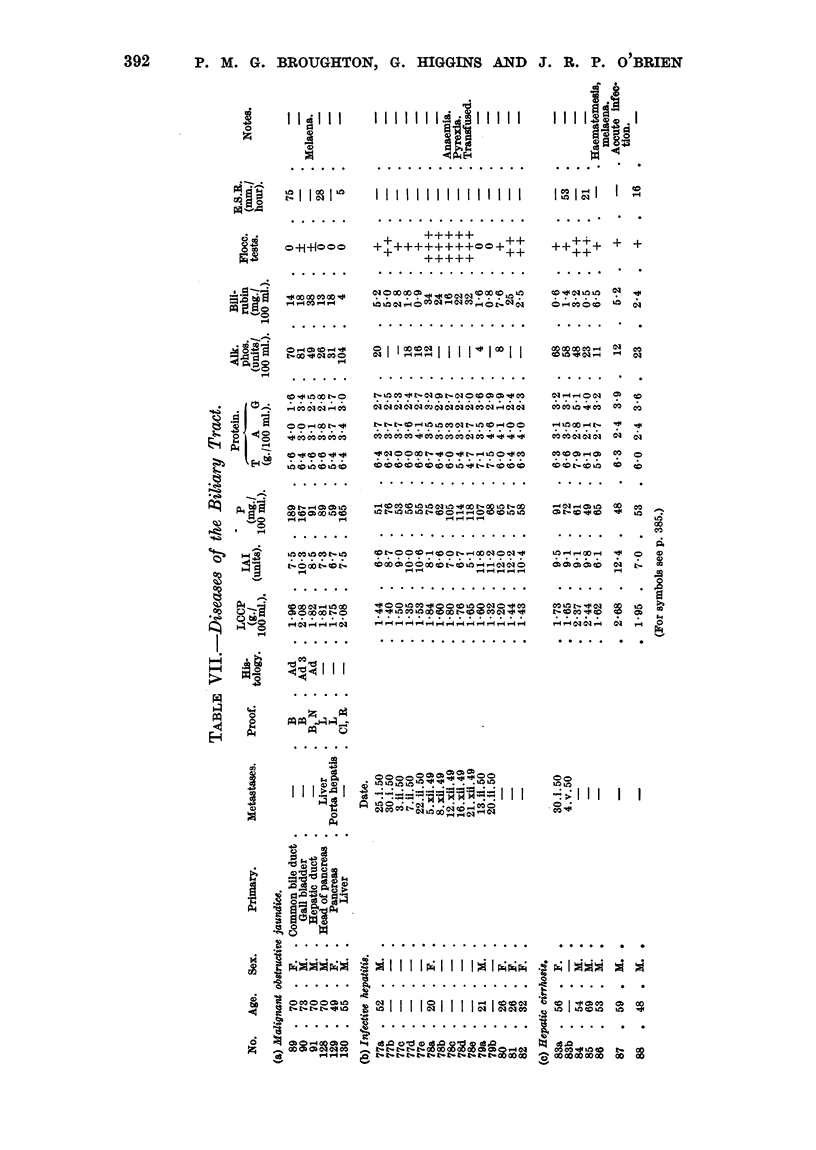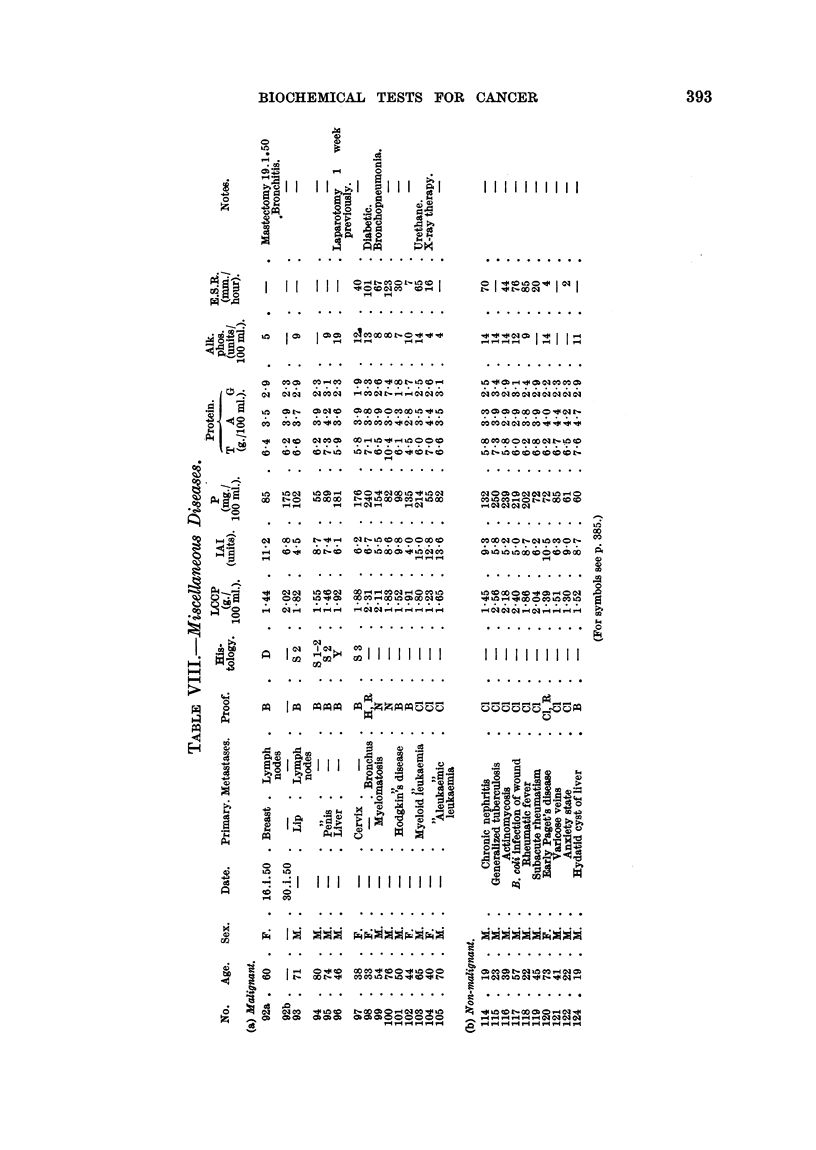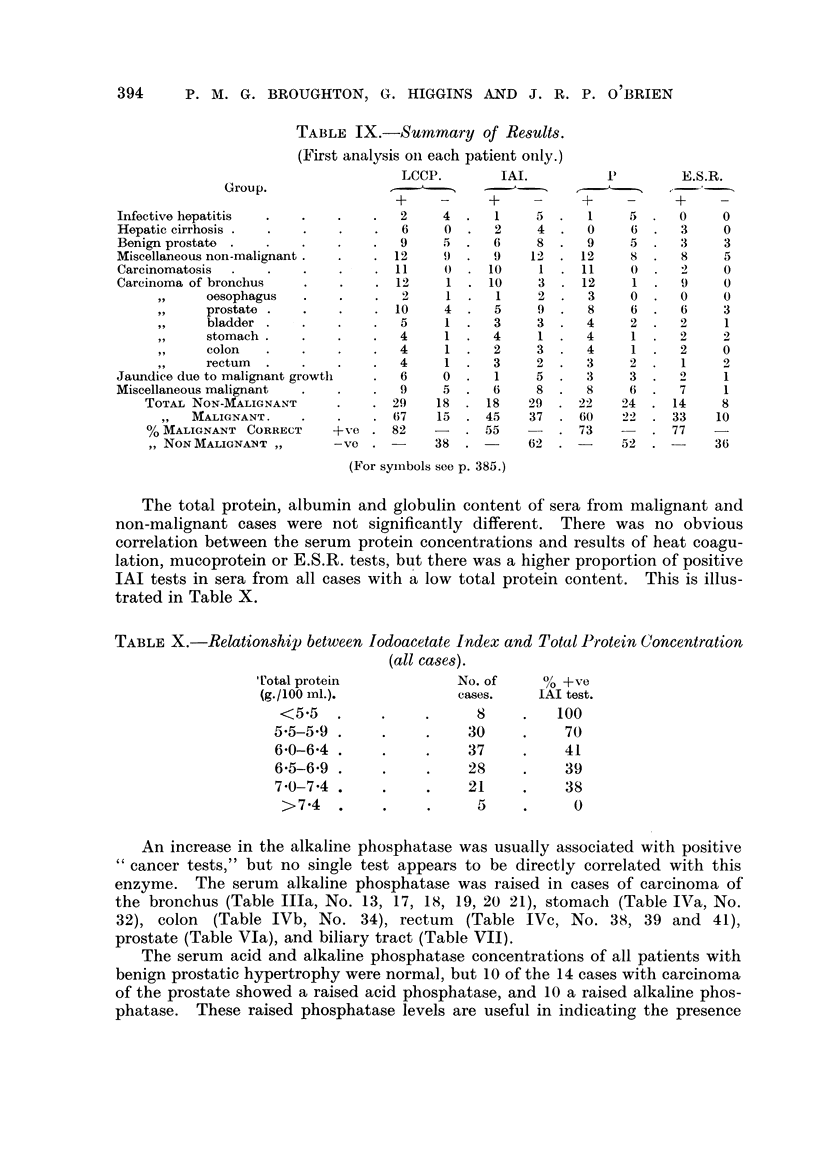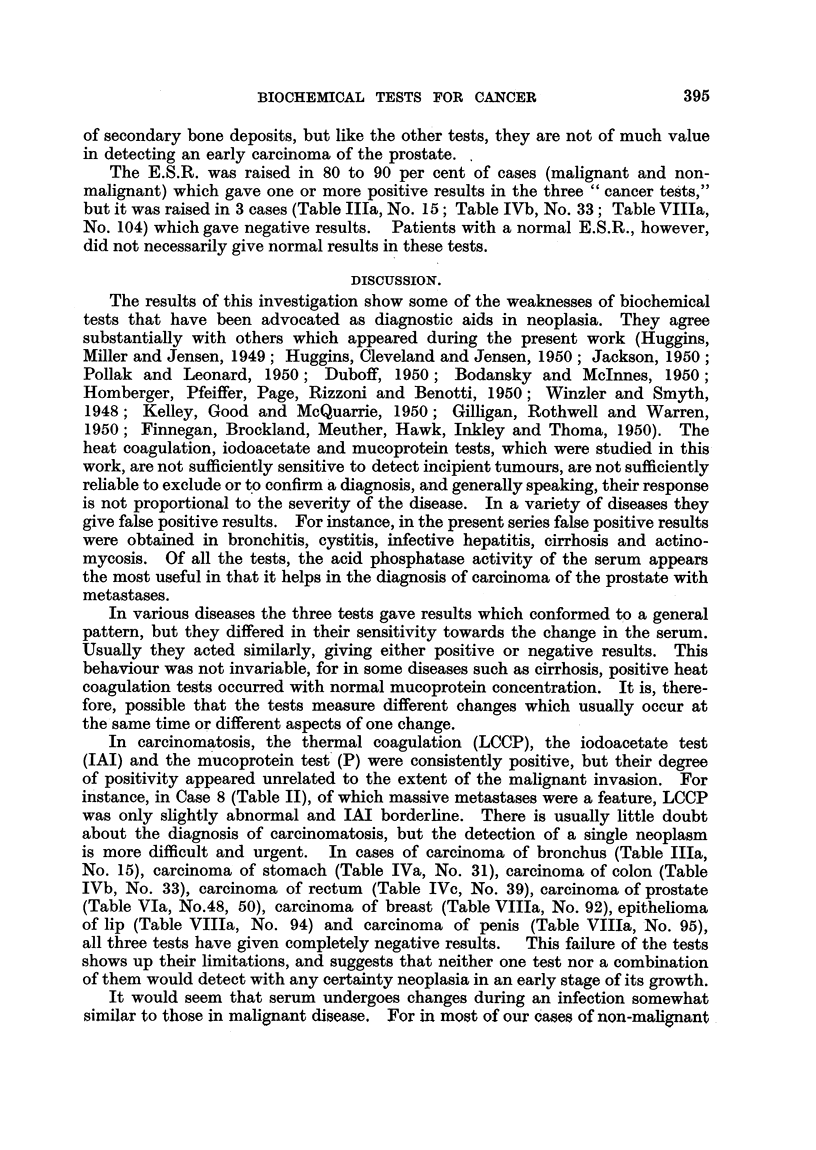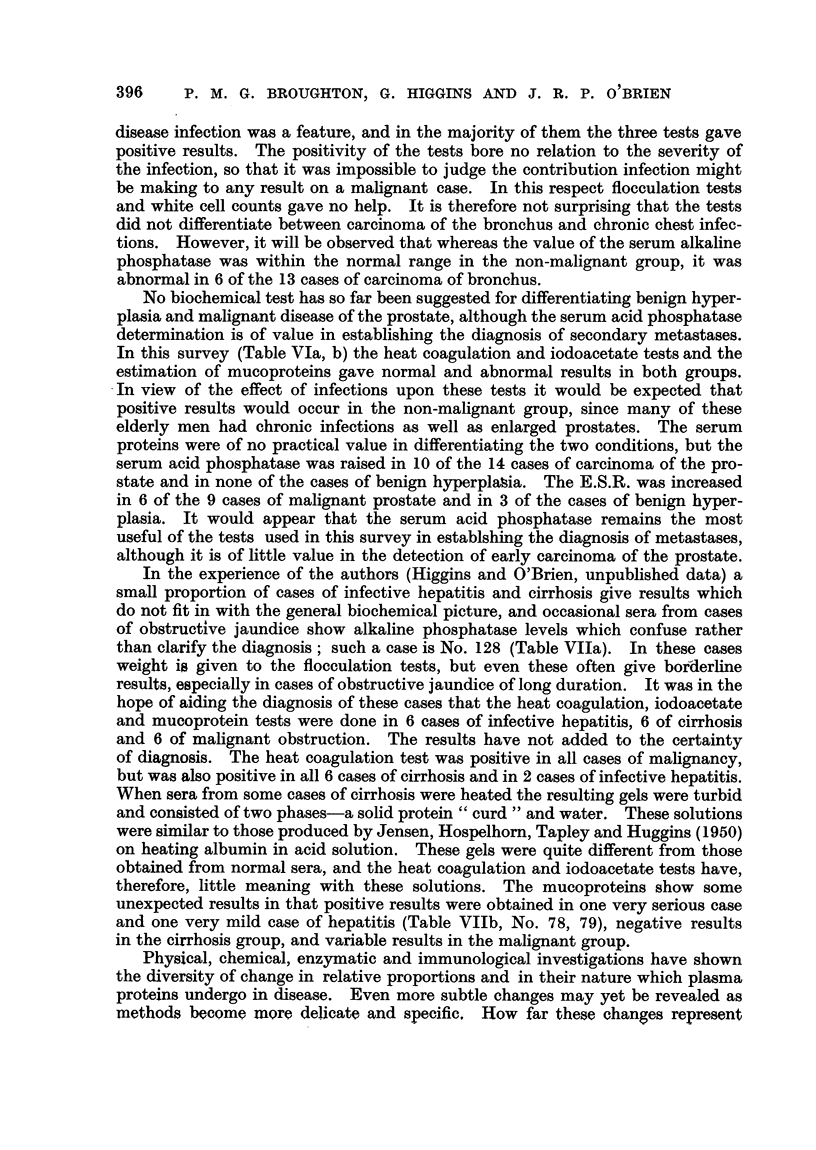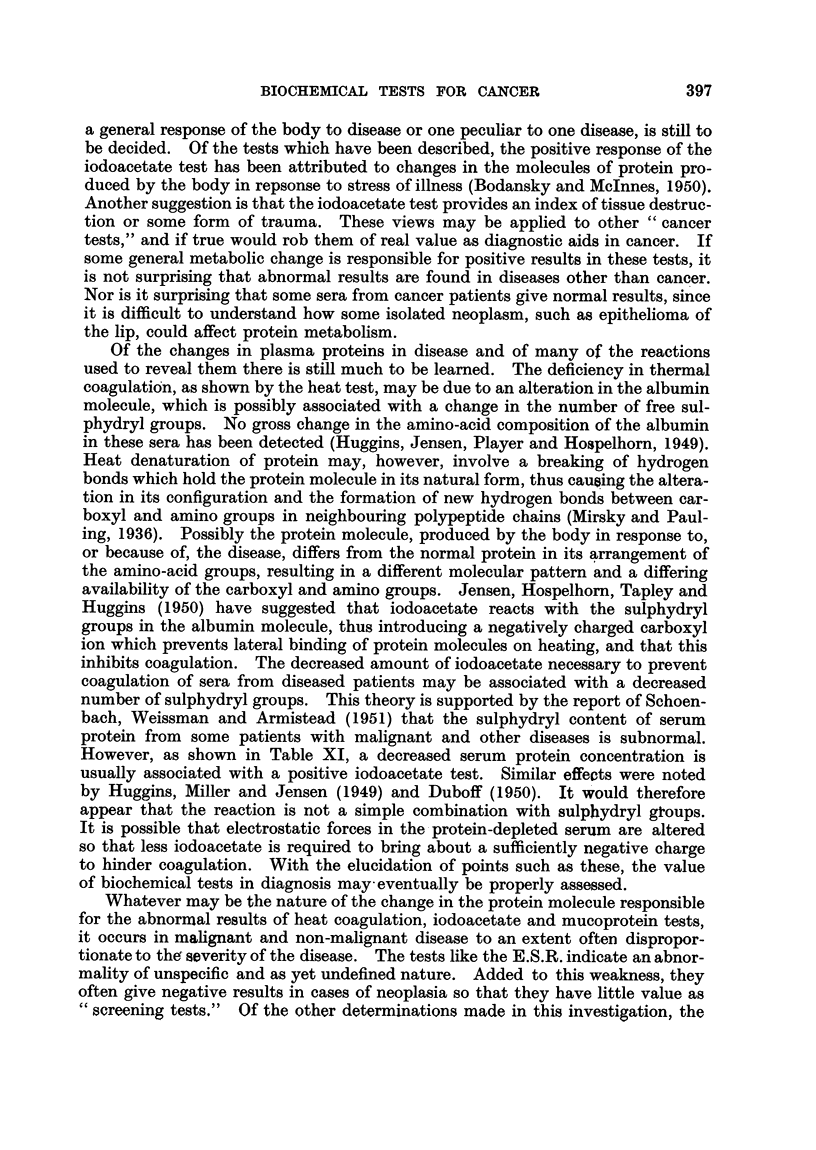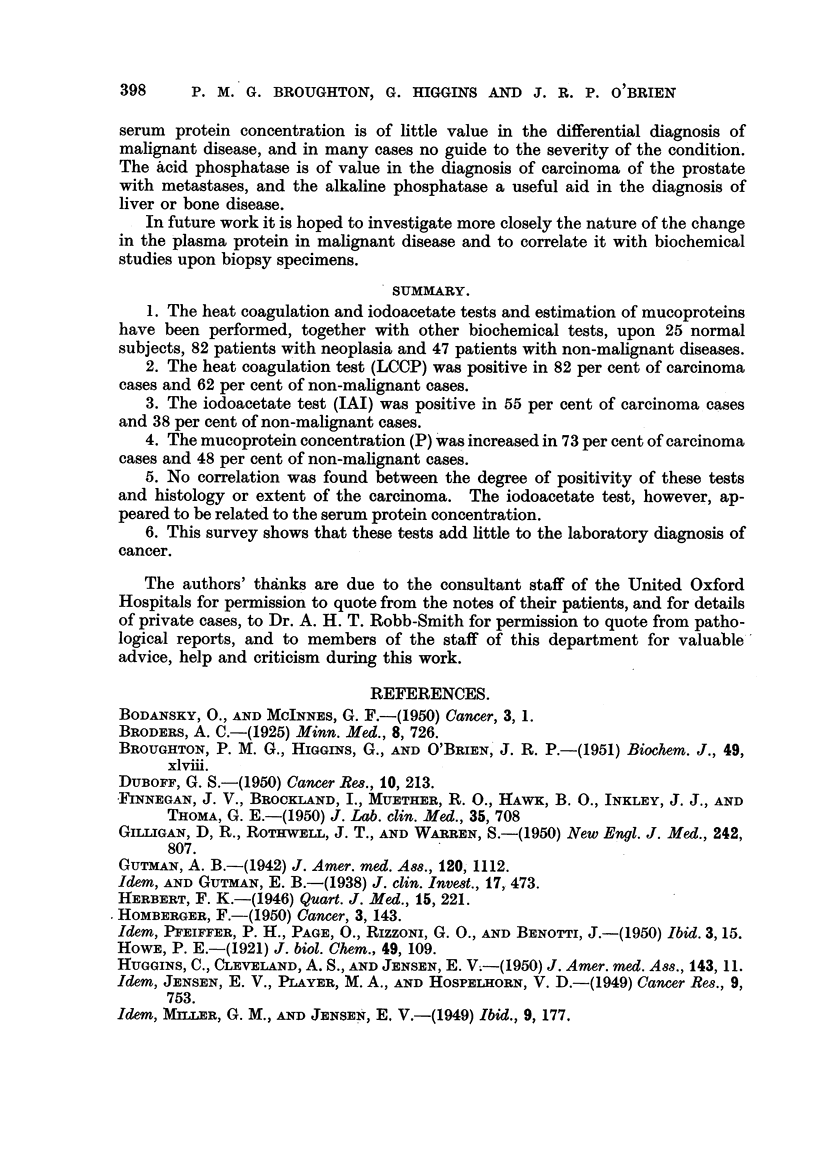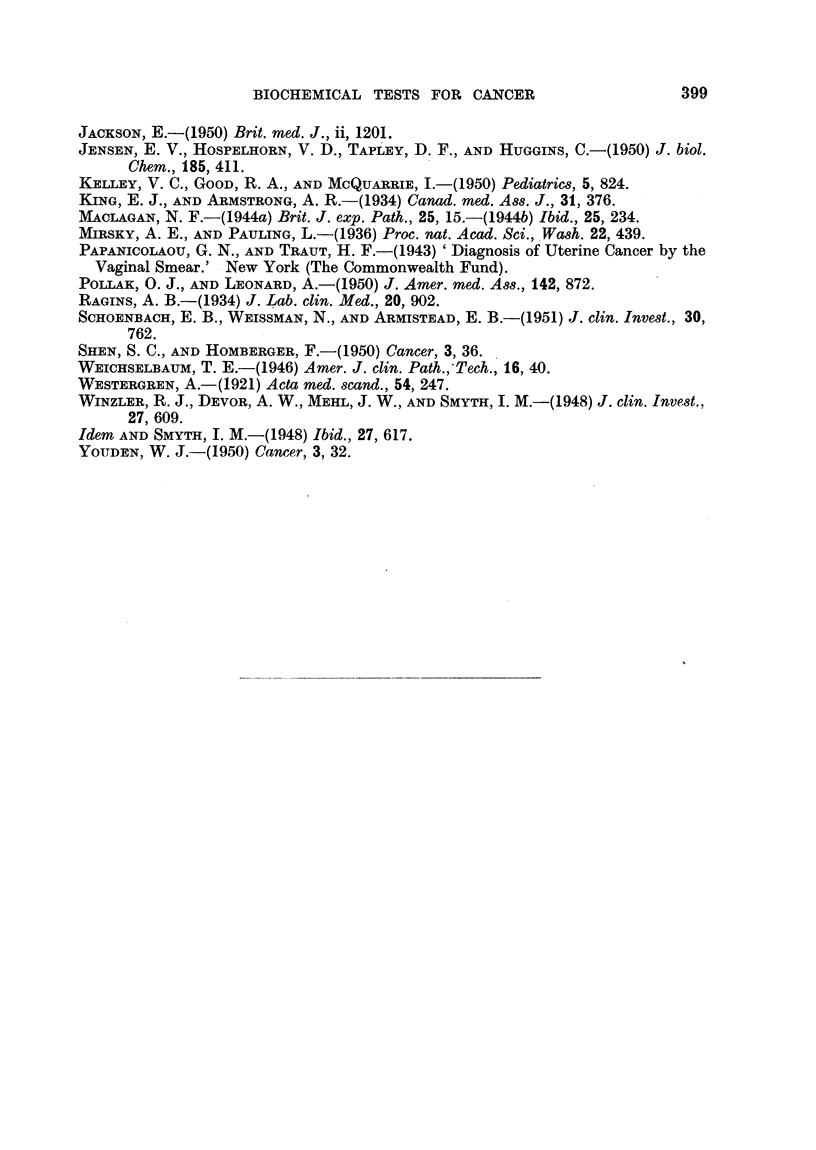# An Evaluation of Some Biochemical Tests for Cancer

**DOI:** 10.1038/bjc.1951.44

**Published:** 1951-12

**Authors:** P. M. G. Broughton, G. Higgins, J. R. P. O'Brien


### AN EVALUATION OF SOME BIOCHEMICAL TESTS FOR CANCER.

P. M. G. BROUGHTON, G. HIGGINS AND J. R. P. O'BRIEN.

*From the Department of Biochemistry, Radcliffe Infirmary, Oxford.*

Received for publication October 5, 1951.

THE eradication of malignant growths by surgery depends largely upon the early diagnosis of the neoplasm. The need for reliable tests for malignancy has been emphasized by Homberger (1950), who has reviewed more than 60 biochemical, biological and cytological " cancer tests " which have been advocated in the last two decades. Some of these, such as the estimation of serum acid phosphatase (Gutman, 1942 ; Herbert, 1946) and the cytological examination of fluids (Papanicolaou and Traut, 1943 ; Shen and Homberger, 1950), are of great value ; others, such as the determination of erythrocyte sedimentation rate (E.S.R.), are not specific for neoplasia.

The growth of malignant tumours involves an abnormal synthesis of tissue which may be reflected in the serum proteins. Recently serious attention has been given to the qualitative and quantitative changes occurring in serum proteins in neoplasia. Huggins and his colleagues (Huggins, Miller and Jensen, 1949 ; Huggins, Jensen, Player and Hospelhorn, 1949 ; Huggins, Cleveland and Jensen, 1950) have shown that the thermal coagulation of serum proteins, and the inhibition of this coagulation by iodoacetate, is altered in neoplastic disease. Winzler and his colleagues (Winzler, Devor, Mehl and Smyth, 1948 ; Winzler and Smyth, 1948) have reported that the mucoproteins in serum may be increased in neoplastic disease. Both groups of authors have, however, demonstrated that these tests are not specific for neoplasia. Several reports (Pollak and Leonard, 1950 ; Bodansky and McInnes, 1950 ; Homberger, Pfeiffer, Page, Rizzoni and Benotti, 1950 ; Gilligan, Rothwell and Warren, 1950 ; Finnegan, Brockland, Muether, Hawk, Inkley and Thoma, 1950) have since been published confirming that some of these tests may not only give positive results in non-malignant cases (i.e., false positive results), but also negative results in malignant cases (i.e., false negative results). Few reports have, however, appeared in which these tests have been used in conjunction with other biochemical tests of known value, such as the estimation of serum acid phosphatase in carcinoma of the prostate and serum alkaline phosphatase in the detection of bone diseases.

This paper records the results of an investigation of the thermal coagulation test, the inhibition of coagulation by iodoacetate and the estimation of serum mucoproteins in 25 normal subjects and 129 patients suffering from malignant neoplasia and other diseases. The serum total protein, albumin and globulin content, the serum phosphatases and E.S.R. have also been estimated for most of the patients and compared with the accepted normal values. A preliminary report of the work was communicated to the Biochemical Society (Broughton, Higgins and O'Brien, 1951).

### METHODS AND MATERIALS.

Patients were chosen, usually before treatment, in whom the diagnosis was proved or likely to be proved. Some with carcinoma, particularly the elderly ones, had complicating diseases, such as chest and urinary infections.

The least concentration of coagulable protein (LCCP) and the iodoacetate index (IAI) were determined by the method of Huggins, Miller and Jensen (1949). In the LCCP test solutions were prepared so that the serum concentration increased in steps of 0·25 ml., and in the IAI test the iodoacetate concentration was increased in steps of 1·5 µM., which were the smallest changes in concentration detectable in our procedure. These solutions were heated in tubes 1 cm. in diameter. In our technique the criterion of coagulation was the formation of a gel sufficiently rigid to slide down the inverted tube after gentle tapping without loss of shape.

Mucoproteins in serum were separated by the method of Winzler, Devor, Mehl and Smyth (1948), and their protein content determined by the biuret method of Weichselbaum (1946).

Serum proteins were determined by the micro-Kjeldahl technique (Howe, 1921). Alkaline phosphatase was determined by the method of King and Armstrong (1934), and acid phosphatase by the method of Gutman and Gutman (1938). The erythrocyte sedimentation rate (E.S.R.) was determined by Westergren's (1921) method. All determinations except the E.S.R. were made on serum, and completed on the day the blood was collected.

The serum flocculation tests used were the thymol turbidity test (Maclagan, 1944*b*), the colloidal gold test (Maclagan, 1944*a*) and the Takata-ara reaction (Ragins, 1934).

### RESULTS.

The following symbols are used in the text and tables :

T = Total protein content of serum (g./100 ml.).
A = Albumin content of serum (g./100 ml.).
G = Globulin content of serum (g./100 ml.).
LCCP = Least concentration of coagulable protein, i.e., the least concentration of serum protein which coagulates on heating (g./100 ml.).
IAI = Iodoacetate index. In this test the end-point is the largest amount of iodoacetate (µM.) which, when added to 0·25 ml. serum, will not inhibit thermal coagulation. Then IAI is this end-point multiplied by 4/T.
P = mg. protein in the mucoprotein present in 100 ml. serum.

| | |
|---|---|
| B = Biopsy. | H = Bronchoscopy. |
| L = Laparotomy. | Ad = Adenocarcinoma. |
| N = Necropsy. | TC = Transitional-celled carcinoma. |
| Cl. = Clinical diagnosis. | S = Squamous-celled carcinoma. |
| R = Radiological diagnosis. | D = Spheroidal-celled carcinoma. |
| M = Malignant cells in pleural fluid. | Y = Leiomyosarcoma. |

The numbers in the histology column refer to Broders' (1925) grading. The diagnosis of malignancy was confirmed by laparotomy, necropsy, histological or radiological examination in 80 cases.

TABLE I.—*Normal Ranges for the Least Concentration of Coagulable Protein, the Iodoacetate Index and Serum Mucoprotein Concentration.*

| Test. | No. of analyses. | Range. |
|---|---|---|
| LCCP . . . | 32 . | 1·19–1·59 g./100 ml. |
| IAI . . . | 30 . | 7·3–13·6 units |
| P . . . . | 32 . | 40–93 mg./100 ml. |

(For symbols see p. 385.)

*Normal subjects.*

Table I shows the range of values for the thermal coagulation and iodoacetate tests and the estimation of mucoprotein obtained in 32 analyses on a group of 11 females and 14 males, of ages ranging from 20 to 40 years (mean age 26).

The normal range for alkaline phosphatase was taken as 4–14 units/100 ml., total protein 6·3–7·8 g./100 ml., albumin 4·0–5·2 g./100 ml., globulin 1·3–2·9 g./100 ml. (Higgins and O'Brien, unpublished data), acid phosphatase 1–5 units/100 ml. (Gutman and Gutman, 1938), and E.S.R. 1–10 mm./hour (Westergren, 1921).

*Hospital patients.*

Tables II to VIII show the values for the thermal coagulation test, iodoacetate index and mucoprotein concentration, together with the serum proteins, of a group of 82 patients with malignant diseases and 47 patients with non-malignant diseases. The malignant group contained 11 cases of carcinomatosis, 16 neoplasia of bronchus and oesophagus, 15 neoplasia of gastro-intestinal tract, 20 neoplasia of prostate and bladder, 6 neoplasia of biliary tract and 14 miscellaneous malignant diseases. The non-malignant group contained 7 cases of chest infection, 14 benign prostatic hyperplasia, 12 hepatic diseases and 14 miscellaneous diseases.

Table IX records the summary of the results given in Tables II to VIII. It will be seen from this table that none of these tests is completely reliable in the detection of malignancy; all tests gave false positive and false negative results. The LCCP test gave 82 per cent correct positive results in the malignant group, but 62 per cent false positives in the non-malignant group. Similarly the IAI gave 55 per cent correct positive results and 38 per cent false positive results, while the mucoprotein concentration was abnormal in 73 per cent of the malignant cases, but also 48 per cent of the non-malignant cases.

The least concentration of coagulable globulin and the actual amount of iodoacetate added have also been investigated but yielded no additional information.

Youden (1950) has proposed an index for assessing the value of diagnostic tests. These indices were calculated for LCCP, IAI and mucoprotein (P), but differences between them were not significant and they have not therefore been reported.

*Correlation of results.*

None of the three tests, LCCP, IAI and P, detected one histological type of neoplasia better than another, and it was impossible from their results to determine whether the more rapidly growing neoplasms produced greater changes in the serum. Their degree of positivity in cases of widespread carcinomatosis was often no greater than that in cases of single neoplasm.

TABLE II.—*Carcinomatosis.*

(Site of primary growth uncertain.)

| No. | Age. | Sex. | Date. | Metastases. | Proof. | Histology. | LCCP (g./100 ml.). | IAI (units). | P (mg./100 ml.). | Protein T (g./100 ml.) | Protein A (g./100 ml.) | Protein G (g./100 ml.) | Alk. phos. (units/100 ml.). | E.S.R. (mm./hour). | Notes |
|---|---|---|---|---|---|---|---|---|---|---|---|---|---|---|---|
| 1a | 62 | M. | 19.xii.49 | Stomach, colon, intestines | L | — | 1·68 | 5·3 | 148 | 5·6 | 3·3 | 2·3 | 6 | — | Laparotomy 3.xii.49. |
| 1b | — | — | 11.i.50 | — | — | — | 1·79 | 3·3 | 176 | 5·5 | 3·0 | 2·5 | 12 | — | — |
| 2a | 41 | F. | 18.i.50 | Liver, colon, skin | B | Ad | 2·56 | 2·8 | 172 | 6·4 | 3·4 | 3·0 | 50 | — | Jaundice, oedema. |
| 2b | — | — | 20.ii.50 | — | — | — | 2·17 | 3·9 | 143 | 6·2 | 3·3 | 2·9 | 90 | — | — |
| 3 | 38 | M. | — | Peritoneum, colon, intestines | B | Ad 2–3 | 2·48 | 5·8 | 179 | 6·2 | 3·8 | 2·4 | — | — | Laparotomy 2 days previously. |
| 4 | 48 | M. | — | Stomach, pancreas, spleen | B | Ad 2–3 | 2·85 | 3·0 | 150 | 6·0 | 3·0 | 3·0 | 5 | 44 | — |
| 5 | 41 | F. | — | Colon, peritoneum | B | Ad 2 | 1·99 | 6·8 | 139 | 5·3 | 3·3 | 2·0 | 8 | — | Ascites. |
| 6 | 58 | M. | — | Stomach, liver, spleen | N | — | 1·71 | 4·2 | 160 | 5·7 | 3·3 | 2·4 | 47 | — | Haematemesis. |
| 7 | 71 | M. | — | Liver, intestine, lungs | B, N | Ad | 2·94 | 1·2 | 212 | 5·1 | 2·8 | 2·3 | 8 | — | Laparotomy 3 days previously. |
| 8 | 47 | M. | — | Oesophagus, lungs, intestines | Cl, R | — | 1·72 | 7·8 | 128 | 6·9 | 3·8 | 3·1 | 19 | 88 | — |
| 9 | 70 | M. | — | Bladder, colon, prostate | B | Ad | 2·75 | 3·0 | 246 | 6·1 | 3·2 | 2·9 | 6 | — | — |
| 10 | 56 | M. | — | Colon, liver, intestines | B | Ad | 2·18 | 7·2 | 262 | 6·7 | 4·0 | 2·7 | 17 | — | — |
| 11 | 70 | M. | — | Stomach, pancreas, bones | N | — | 2·00 | 6·3 | 240 | 5·7 | 3·1 | 2·6 | 19 | — | Coronary thrombosis. |

(For symbols see p. 385.)

TABLE III.—*Diseases of Bronchus and Oesophagus.*

| No. | Age. | Sex. | Date. | Metastases. | Proof. | Histology. | LCCP (g./100 ml.). | IAI (units). | P (mg./100 ml.). | Protein T (g./100 ml.) | Protein A | Protein G | Alk. phos. (units/100 ml.). | E.S.R. (mm./hour). | Notes. |
|---|---|---|---|---|---|---|---|---|---|---|---|---|---|---|---|
| *(a) Carcinoma of bronchus.* | | | | | | | | | | | | | | | |
| 12a | 48 | M. | 8.xii.49 | Kidneys, heart | M, N | — | 1·79 | 6·5 | 162 | 5·5 | 3·7 | 1·8 | — | 70 | Hepatic cirrhosis. |
| 12b | — | — | 1.ii.50 | Adrenal | — | — | 2·33 | 6·8 | 193 | 6·2 | 3·6 | 2·6 | 10 | — | — |
| 13a | 38 | M. | 2.i.50 | Liver, spine | Cl, R | — | 2·66 | 3·1 | 258 | 5·9 | 3·4 | 2·5 | 44 | 66 | Pleural effusion. |
| 13b | — | — | 16.i.50 | — | — | — | 3·00 | 2·0 | 231 | 6·0 | 3·3 | 2·7 | 35 | 112 | — |
| 14 | 55 | M. | — | — | B | S3 | 2·28 | 5·1 | 216 | 7·0 | 3·4 | 3·6 | 11 | 124 | — |
| 15 | 63 | M | — | — | M, R | — | 1·50 | 12·0 | 90 | 6·0 | 3·5 | 2·5 | 9 | 35 | Pleural effusion. |
| 16 | 51 | M. | — | — | H, R | — | 1·93 | 6·2 | 139 | 7·0 | 4·3 | 2·7 | 13 | — | — |
| 17 | 67 | M. | — | — | H, R | — | 1·75 | 10·8 | 125 | 7·8 | 3·8 | 4·0 | 18 | — | — |
| 18 | 60 | M. | — | — | B | S3 | 2·75 | 1·0 | 252 | 6·1 | 2·8 | 3·3 | 21 | 56 | Pleural effusion. |
| 19 | 54 | M. | — | Liver, skin | Cl, R | — | 1·95 | 5·8 | 167 | 5·2 | 3·0 | 2·2 | 48 | — | — |
| 20 | 76 | M. | — | — | Cl, R | — | 2·24 | 4·3 | 224 | 6·9 | 3·3 | 3·6 | 17 | 84 | Bronchitis. |
| 21 | 60 | M. | — | Liver, brain | Cl, R | — | 1·84 | 7·2 | 164 | 6·7 | 3·4 | 3·3 | 59 | 70 | — |
| 22 | 52 | M. | — | — | H, Cl | — | 1·87 | 8·8 | 125 | 7·5 | 3·8 | 3·7 | 12 | 30 | Bronchopneumonia. |
| 23 | 68 | M. | — | Oesophagus | N | — | 2·24 | 5·6 | 174 | 6·4 | 3·2 | 3·2 | 5 | 82 | Bronchopneumonia, hypertension. |
| 24 | 71 | M. | — | — | Cl | — | 2·76 | 2·1 | 241 | 5·8 | 3·7 | 2·1 | 13 | — | Dermatitis. |
| *(b) Carcinoma of oesophagus.* | | | | | | | | | | | | | | | |
| 25 | 73 | M. | — | — | B | S2 | 1·68 | 8·1 | 107 | 6·7 | 4·4 | 2·3 | 4 | — | Cerebral thrombosis. |
| 26 | 71 | F. | — | — | B, N | S2 | 1·65 | 8·2 | 126 | 6·6 | 3·6 | 3·0 | 6 | — | — |
| 27 | 64 | M. | — | — | B, N | S2 | 1·51 | 6·5 | 160 | 5·5 | 3·5 | 2·0 | 8 | — | — |

| No. | Age. | Sex. | Disease. | Proof. | LCCP (g./100 ml.). | IAI (units). | P (mg./100 ml.). | Proteins T (g./100 ml.) | Proteins A | Proteins G | Alk. phos. (units/100 ml.). | E.S.R. (mm./hour). |
|---|---|---|---|---|---|---|---|---|---|---|---|---|
| *(c) Chest infections.* | | | | | | | | | | | | |
| 106 | 56 | M. | Bronchopneumonia | Cl | 2·22 | 6·2 | 153 | 6·8 | 3·8 | 3·0 | — | — |
| 107 | 57 | M. | Head injury + pneumonia | Cl, N | 1·62 | 8·1 | — | 5·9 | 4·4 | 1·5 | — | — |
| 108 | 69 | M. | Chronic bronchitis | Cl | 2·12 | 6·5 | 135 | 6·5 | 3·3 | 3·2 | — | 3 |
| 109 | 68 | M. | " " | Cl | 1·75 | 6·8 | 122 | 7·0 | 3·2 | 3·8 | 11 | 31 |
| 110 | 19 | M. | Bronchiectasis | Cl | 1·31 | 9·3 | 64 | 5·8 | 4·0 | 1·8 | 9 | 2 |
| 111 | — | F. | " | Cl | 1·28 | 8·4 | 60 | 6·4 | 4·0 | 2·4 | 10 | — |
| 112 | 54 | M. | Bronchopneumonia + pyelonephritis | Cl, L | 2·22 | 8·1 | 201 | 7·4 | 3·6 | 3·8 | 10 | 77 |

(For symbols see p. 385.)

TABLE IV.—*Diseases of the Gastro-intestinal Tract.*

| No. | Age. | Sex. | Date. | Metastases. | Proof. | His-tology. | LCCP (g./100 ml.). | IAI (units). | P (mg./100 ml.). | Protein. (g./100 ml.) T | A | G | Alk. phos. (units/100 ml.). | E.S.R. (mm./hour). | Notes. |
|---|---|---|---|---|---|---|---|---|---|---|---|---|---|---|---|
| (a) *Carcinoma of stomach.* | | | | | | | | | | | | | | | |
| 28a | 51 | F. | 13.xii.49 | — | B | Ad 3–4 | 1·85 | 6·3 | 188 | 5·7 | 3·7 | 2·0 | 3 | 40 | Gastrectomy 13.xii.49. |
| 28b | — | — | 21.xii.49 | — | — | — | 2·29 | 3·9 | 212 | 6·1 | 3·7 | 2·4 | — | — | — |
| 28c | — | — | 11.i.50 | — | — | — | 1·81 | 7·3 | 123 | 6·6 | 4·1 | 2·5 | 10 | — | — |
| 29a | 61 | M. | 13.i.50 | Lymph nodes, colon, pancreas | B | Ad 3 | 1·87 | 6·0 | 146 | 5·0 | 3·1 | 1·9 | 11 | — | Gastrectomy 16.i.50. |
| 29b | — | — | 27.i.50 | — | — | — | 2·79 | 3·4 | 246 | 5·3 | 3·2 | 2·1 | 12 | — | — |
| 30 | 61 | M. | — | *nil* | N | — | 3·26 | 2·9 | 172 | 6·2 | 3·4 | 2·8 | — | 8 | Bronchopneumonia. |
| 31 | 65 | M. | — | — | B | Ad 2–3 | 1·49 | 9·7 | 80 | 6·8 | 4·2 | 2·6 | 14 | 10 | Bronchitis. |
| 32 | 48 | F. | — | Liver, lymph nodes | Cl, R | — | 1·81 | 6·3 | 126 | 5·7 | 3·5 | 2·2 | 38 | 37 | — |
| (b) *Carcinoma of colon.* | | | | | | | | | | | | | | | |
| 33 | 58 | M. | — | Lymph nodes | B | Ad | 1·14 | 10·5 | 74 | 5·7 | 4·0 | 1·7 | 4 | 14 | — |
| 34 | 59 | F. | — | Lymph nodes, intestines | B | Ad | 1·87 | 9·7 | 185 | 6·8 | 4·6 | 2·2 | 18 | — | — |
| 35 | 69 | F. | — | Liver | B | Ad 3 | 1·80 | 7·5 | 117 | 7·2 | 4·2 | 3·0 | 9 | — | Laparotomy 4 days previously. |
| 36 | 39 | F. | — | Ureter | B | Ad 2–3 | 1·83 | 6·9 | 147 | 6·1 | 3·5 | 2·6 | — | 55 | Colostomy 17 days previously. |
| 37 | 66 | F. | — | Peritoneum | L | — | 2·0 | 6·3 | 242 | 5·7 | 3·7 | 2·0 | 4 | — | — |
| (c) *Carcinoma of rectum.* | | | | | | | | | | | | | | | |
| 38 | 62 | F. | — | Lymph nodes, colon | B | Ad 2–3 | 1·89 | 6·7 | 180 | 6·3 | 3·7 | 2·6 | 18 | — | — |
| 39 | 60 | M. | — | — | B | Ad 2–3 | 1·38 | 9·8 | 54 | 5·5 | 4·1 | 1·4 | 16 | 10 | Arteriosclerosis. |
| 40 | 41 | M. | — | — | L | — | 1·62 | 4·3 | 112 | 6·5 | 3·8 | 2·7 | 7 | 38 | — |
| 41a | 59 | F. | 19.xii.49 | Liver, lungs | B, N | Ad 2 | 2·06 | 5·1 | 248 | 5·9 | 3·3 | 2·6 | 47 | — | — |
| 41b | — | — | 11.i.50 | — | — | — | 2·65 | 1·1 | 236 | 5·3 | 3·0 | 2·3 | 58 | — | — |
| 42 | 70 | M. | — | — | B | Ad 2–3 | 1·71 | 7·4 | 87 | 5·7 | 2·4 | 3·3 | — | 10 | — |
| (d) *Non-malignant.* | | | | | | | | | | | | | | | |
| 123 | 69 | M. | — | Duodenal ulcer | N | — | 1·55 | 8·7 | 126 | 6·9 | 3·8 | 3·1 | 7 | — | — |

(For symbols see p. 385.)

TABLE V.—*Diseases of Bladder.*

| No. | Age. | Sex. | Date. | Proof. | Histology. | LCCP (g./100 ml.). | IAI (units). | P (mg./100 ml.). | Protein (g./100 ml.) T | Protein A | Protein G | Alk. phos. (units/100 ml.). | E.S.R. (mm./hour). | Notes. |
|---|---|---|---|---|---|---|---|---|---|---|---|---|---|---|
| (a) *Carcinoma.* | | | | | | | | | | | | | | |
| 57a | 55 | M. | 17.i.50 | B | TC2 | 1·57 | 5·1 | 47 | 7·0 | 4·3 | 2·7 | 7 | — | Diabetes, asthma. |
| 57b | — | — | 3.ii.50 | — | — | 1·76 | 7·6 | 132 | 6·3 | 4·0 | 2·3 | 10 | — | Cystectomy 20.i.50. |
| 58 | 65 | M. | — | B | TC | 1·86 | 7·7 | 195 | 6·2 | 3·8 | 2·4 | 7 | 60 | Acute infection. |
| 59 | 69 | M. | — | B | TC1 | 2·16 | 6·7 | 132 | 7·2 | 3·7 | 3·5 | 13 | 32 | Chronic cough. |
| 60 | 71 | M. | — | L | — | 1·80 | 7·5 | 62 | 7·2 | 4·1 | 3·1 | 7 | 7 | — |
| 61 | 66 | M. | — | B, N | S 3–4 | 1·95 | 6·0 | 148 | 6·0 | 3·9 | 2·1 | 12 | — | — |
| 62 | 50 | F. | — | B | S 2–3 | 1·80 | 8·0 | 159 | 6·0 | 3·9 | 2·1 | 8 | — | Rheumatoid arthritis. |
| (b) *Non-malignant.* | | | | | | | | | | | | | | |
| 113a | 45 | M. | 20.i.50 | Cl, B | — | 1·78 | 8·5 | 159 | 7·1 | 4·1 | 3·0 | 9 | — | Pyelonephritis + chronic cystitis. |
| 113b | — | — | 21.ii.50 | — | — | 2·51 | 4·1 | 285 | 5·9 | 3·5 | 2·4 | — | — | — |
| 125 | 62 | M. | — | B | — | 1·45 | 8·3 | 55 | 6·5 | 3·6 | 2·9 | 6 | 2 | Non-malignant transitional celled papilloma of bladder. |
| 126 | 72 | M. | — | Cl | — | 1·95 | 4·6 | 125 | 5·2 | 3·7 | 1·5 | 5 | 24 | Cystitis; emphysema; arteriosclerosis. |

(For symbols see p. 385.)

### TABLE VI.—*Diseases of Prostate.*

| No. | Age. | Date. | Metastases. | Proof. | Histology. | LCCP (g./100 ml.). | IAI (units). | P (mg./100 ml.). | Protein T (g./100 ml.) | Protein A (g./100 ml.) | Protein G (g./100 ml.) | Acid phos. (units/100 ml.) | Alk. phos. (units/100 ml.) | E.S.R. (mm./hour). | Notes. |
|---|---|---|---|---|---|---|---|---|---|---|---|---|---|---|---|
| *(a) Carcinoma of prostate.* | | | | | | | | | | | | | | | |
| 43 | 69 | — | Liver, lymph nodes | B, N | Ad 2 | 2·21 | 4·1 | 140 | 5·9 | 3·0 | 2·9 | 70 | 23 | 27 | After stilboestrol treatment, bronchopneumonia. |
| 44 | 67 | — | Spine, pelvis | B | Ad 1–2 | 2·03 | 6·7 | 116 | 5·4 | 3·5 | 1·9 | 7 | 67 | 35 | After stilboestrol treatment. |
| 45 | 71 | — | — | B | 4 | 1·48 | 7·1 | 87 | 5·9 | 3·7 | 2·2 | 7 | 11 | 10 | " " " |
| 46 | 63 | — | — | B | Ad | 2·19 | 7·4 | 109 | 7·3 | 3·7 | 3·6 | 3 | 15 | — | " " " |
| 47 | 66 | — | — | B | Ad | 1·73 | 8·7 | 83 | 6·9 | 3·0 | 3·9 | 21 | 18 | 34 | — |
| 48 | 68 | — | — | B | Ad | 1·48 | 11·3 | 92 | 7·4 | 4·5 | 2·9 | 3 | 9 | — | Chest infection. |
| 49 | 71 | — | — | B | Ad | 1·38 | 9·8 | 152 | 6·5 | 4·0 | 2·5 | 12 | 15 | 36 | — |
| 50 | 53 | — | Pelvis | Cl, R | — | 1·48 | 8·2 | 53 | 6·6 | 3·4 | 3·2 | 2 | 6 | 2 | After stilboestrol treatment, chest infection. |
| 51 | 68 | — | Spine | Cl, R | — | 1·85 | 6·3 | 111 | 5·7 | 3·7 | 2·0 | 33 | 25 | — | — |
| 52 | 76 | — | Chest, pelvis, spine | Cl, R | — | 1·79 | 7·6 | 195 | 5·5 | 3·6 | 1·9 | 101 | 88 | 33 | Bleeding duodenal ulcer. |
| 53a | 62 | 19.xii.49 | Pelvis | Cl, R | — | 1·73 | 8·6 | 85 | 6·3 | 4·3 | 2·0 | 12 | 58 | — | After stilboestrol treatment. |
| 53b | — | 6.ii.50 | — | — | — | 1·98 | 7·9 | 113 | 6·1 | 4·0 | 2·1 | 11 | 70 | — | — |
| 54a | 73 | 9.xii.49 | Pelvis, ribs, spine | Cl, R | — | 1·80 | 7·5 | 125 | 7·2 | 4·6 | 2·6 | 63 | 53 | — | Stilboestrol treatment from 4.xii.49. |
| 54b | — | 16.i.50 | — | — | — | 1·55 | 5·8 | 55 | 6·2 | 4·2 | 2·0 | 17 | 74 | — | — |
| 55 | 81 | — | — | B | — | 2·10 | 6·0 | 194 | 6·0 | 3·1 | 2·9 | 6 | 60 | 62 | — |
| 56 | 68 | — | — | Cl | — | 1·65 | 7·3 | 61 | 6·6 | 3·7 | 2·9 | 1 | 4 | 8 | After stilboestrol treatment. |

| No. | Age. | Date. | Proof. | LCCP (g./100 ml.). | IAI (units). | P (mg./100 ml.). | Protein T (g./100 ml.) | Protein A (g./100 ml.) | Protein G (g./100 ml.) | E.S.R. (mm./hour). | Notes. |
|---|---|---|---|---|---|---|---|---|---|---|---|
| *(b) Benign prostatic hypertrophy.* | | | | | | | | | | | |
| 63 | 54 | — | Cl | 1·60 | 11·8 | 56 | 7·1 | 4·3 | 2·8 | 3 | Urine infection. |
| 64 | 76 | — | B | 1·19 | 9·7 | 82 | 6·8 | 3·9 | 2·9 | — | Bronchitis. |
| 65 | 63 | — | B | 1·40 | 9·4 | 44 | 7·0 | 4·1 | 2·9 | — | — |
| 66 | 78 | — | B | 1·35 | 12·0 | 130 | 6·0 | 4·2 | 2·8 | — | — |
| 67 | 89 | — | B, N | 1·74 | 6·2 | 138 | 5·8 | 3·2 | 2·6 | 62 | Bronchitis. |
| 68 | 75 | — | B | 2·74 | 5·8 | 267 | 7·3 | 3·7 | 3·6 | — | — |
| 69 | 62 | — | B | 1·63 | 10·8 | 56 | 6·5 | 4·2 | 2·3 | 8 | — |
| 70 | 66 | — | B | 1·24 | 10·6 | 48 | 6·2 | 4·0 | 2·2 | 2 | — |
| 71 | 74 | — | B | 2·18 | 6·3 | 274 | 6·7 | 3·9 | 2·8 | — | — |
| 72 | 75 | — | B | 1·53 | 8·5 | 104 | 6·1 | 4·0 | 2·1 | 11 | — |
| 73 | 69 | — | N | 1·85 | 9·7 | 134 | 7·4 | 4·5 | 2·9 | — | Pneumonia. |
| 74a | 76 | 1.ii.50 | B | 2·03 | 6·2 | 151 | 5·8 | 3·4 | 2·4 | — | Cystotomy 30.i.50. |
| 74b | — | 22.ii.50 | — | 2·10 | 5·3 | 99 | 5·6 | 3·3 | 2·3 | — | Prostatectomy 17.ii.50. |
| 75 | 81 | — | Cl | 2·10 | 4·3 | 151 | 5·6 | 3·1 | 2·5 | 48 | Bronchopneumonia. |
| 76 | 83 | — | Cl | 1·98 | 4·9 | 147 | 6·1 | 3·9 | 2·2 | — | Cystitis. |

The acid and alkaline phosphatases on all these patients were normal. (For symbols see p. 385.)

TABLE VII.—*Diseases of the Biliary Tract.*

| No. | Age. | Sex. | Primary. | Metastases. | Proof. | Histology. | LCCP (g./100 ml.). | IAI (units). | P (mg./100 ml.). | Protein. T (g./100 ml.) | Protein. A (g./100 ml.) | Protein. G (g./100 ml.) | Alk. phos. (units/100 ml.). | Bilirubin (mg./100 ml.). | Flocc. tests. | E.S.R. (mm./hour). | Notes. |
|---|---|---|---|---|---|---|---|---|---|---|---|---|---|---|---|---|---|
| (a) *Malignant obstructive jaundice.* | | | | | | | | | | | | | | | | | |
| 89 | 70 | F. | Common bile duct | — | B | Ad | 1·96 | 7·5 | 189 | 5·6 | 4·0 | 1·6 | 70 | 14 | 0 | 75 | — |
| 90 | 73 | M. | Gall bladder | — | B | Ad 3 | 2·08 | 10·3 | 167 | 6·4 | 3·0 | 3·4 | 81 | 18 | ± | — | — |
| 91 | 70 | M. | Hepatic duct | — | B, N | Ad | 1·82 | 8·5 | 91 | 5·6 | 3·1 | 2·5 | 49 | 38 | ± | — | Melaena. |
| 128 | 70 | M. | Head of pancreas | Liver | L | — | 1·81 | 7·3 | 89 | 6·6 | 3·8 | 2·8 | 26 | 13 | 0 | 28 | — |
| 129 | 49 | F. | Pancreas | Porta hepatis | L | — | 1·75 | 6·7 | 59 | 5·4 | 3·7 | 1·7 | 31 | 18 | 0 | — | — |
| 130 | 55 | M. | Liver | — | Cl, R | — | 2·08 | 7·5 | 165 | 6·4 | 3·4 | 3·0 | 104 | 4 | 0 | 5 | — |
| (b) *Infective hepatitis.* | | | | Date. | | | | | | | | | | | | | |
| 77a | 52 | M. | | 25.i.50 | | | 1·44 | 6·6 | 51 | 6·4 | 3·7 | 2·7 | 20 | 5·2 | + | — | — |
| 77b | — | — | | 30.i.50 | | | 1·40 | 8·7 | 76 | 6·2 | 3·7 | 2·5 | — | 5·0 | ++ | — | — |
| 77c | — | — | | 3.ii.50 | | | 1·50 | 9·0 | 53 | 6·0 | 3·7 | 2·3 | — | 2·8 | + | — | — |
| 77d | — | — | | 7.ii.50 | | | 1·35 | 10·0 | 56 | 6·0 | 3·6 | 2·4 | 18 | 1·8 | + | — | — |
| 77e | — | — | | 22.ii.50 | | | 1·53 | 10·6 | 55 | 6·8 | 4·1 | 2·7 | 16 | 0·9 | + | — | — |
| 78a | 20 | F. | | 5.xii.49 | | | 1·84 | 8·1 | 75 | 6·7 | 3·5 | 3·2 | 12 | 34 | +++ | — | — |
| 78b | — | — | | 8.xii.49 | | | 1·60 | 6·6 | 62 | 6·4 | 3·5 | 2·9 | — | 24 | +++ | — | — |
| 78c | — | — | | 12.xii.49 | | | 1·80 | 7·0 | 105 | 6·0 | 3·3 | 2·7 | — | 16 | +++ | — | Anaemia. |
| 78d | — | — | | 16.xii.49 | | | 1·76 | 6·7 | 114 | 5·4 | 3·2 | 2·2 | — | 22 | +++ | — | Pyrexia. |
| 78e | — | — | | 21.xii.49 | | | 1·65 | 5·1 | 118 | 4·7 | 2·7 | 2·0 | — | 32 | +++ | — | Transfused. |
| 79a | 21 | M. | | 13.ii.50 | | | 1·60 | 11·8 | 107 | 7·1 | 3·5 | 3·6 | 4 | 1·6 | 0 | — | — |
| 79b | — | — | | 20.ii.50 | | | 1·32 | 11·2 | 68 | 7·5 | 4·6 | 2·9 | — | 0·8 | 0 | — | — |
| 80 | 26 | F. | | — | | | 1·20 | 12·0 | 65 | 6·0 | 4·1 | 1·9 | 8 | 7·6 | + | — | — |
| 81 | 26 | F. | | — | | | 1·44 | 12·2 | 57 | 6·4 | 4·0 | 2·4 | — | 25 | ++ | — | — |
| 82 | 32 | F. | | — | | | 1·43 | 10·4 | 58 | 6·3 | 4·0 | 2·3 | — | 2·5 | ++ | — | — |
| (c) *Hepatic cirrhosis.* | | | | | | | | | | | | | | | | | |
| 83a | 56 | F. | | 30.i.50 | | | 1·73 | 9·5 | 91 | 6·3 | 3·1 | 3·2 | 68 | 0·6 | + | — | — |
| 83b | — | — | | 4.v.50 | | | 1·65 | 9·1 | 72 | 6·6 | 3·5 | 3·1 | 58 | 1·4 | + | 53 | — |
| 84 | 54 | M. | | — | | | 2·37 | 9·1 | 61 | 7·9 | 2·8 | 5·1 | 48 | 3·2 | ++ | — | — |
| 85 | 69 | M. | | — | | | 2·44 | 9·8 | 49 | 6·1 | 2·1 | 4·0 | 23 | 0·5 | ++ | 21 | — |
| 86 | 53 | M. | | — | | | 1·62 | 6·1 | 65 | 5·9 | 2·7 | 3·2 | 11 | 6·5 | + | — | Haematemesis, melaena. |
| 87 | 59 | M. | | — | | | 2·68 | 12·4 | 48 | 6·3 | 2·4 | 3·9 | 12 | 5·2 | + | — | Accute infection. |
| 88 | 48 | M. | | — | | | 1·95 | 7·0 | 53 | 6·0 | 2·4 | 3·6 | 23 | 2·4 | + | 16 | — |

(For symbols see p. 385.)

TABLE VIII.—*Miscellaneous Diseases.*

| No. | Age. | Sex. | Date. | Primary. | Metastases. | Proof. | Histology. | LCCP (g./100 ml.). | IAI (units). | P (mg./100 ml.). | Protein (g./100 ml.) T | A | G | Alk. phos. (units/100 ml.). | E.S.R. (mm./hour). | Notes. |
|---|---|---|---|---|---|---|---|---|---|---|---|---|---|---|---|---|
| (a) *Malignant.* | | | | | | | | | | | | | | | | |
| 92a | 60 | F. | 16.i.50 | Breast | Lymph nodes | B | D | 1·44 | 11·2 | 85 | 6·4 | 3·5 | 2·9 | 5 | — | Mastectomy 19.1.50. Bronchitis. |
| 92b | — | — | 30.i.50 | — | — | — | — | 2·02 | 6·8 | 175 | 6·2 | 3·9 | 2·3 | — | — | — |
| 93 | 71 | M. | — | Lip | Lymph nodes | B | S 2 | 1·82 | 4·5 | 102 | 6·6 | 3·7 | 2·9 | 9 | — | — |
| 94 | 80 | M. | — | „ | — | B | S 1–2 | 1·55 | 8·7 | 55 | 6·2 | 3·9 | 2·3 | — | — | — |
| 95 | 74 | M. | — | Penis | — | B | S 2 | 1·46 | 7·4 | 89 | 7·3 | 4·2 | 3·1 | 9 | — | — |
| 96 | 46 | M. | — | Liver | — | B | Y | 1·92 | 6·1 | 181 | 5·9 | 3·6 | 2·3 | 19 | — | Laparotomy 1 week previously. |
| 97 | 38 | F. | — | Cervix | — | B | S 3 | 1·88 | 6·2 | 176 | 5·8 | 3·9 | 1·9 | 12 | 40 | — |
| 98 | 33 | F. | — | — | Bronchus | H, R | — | 2·31 | 6·7 | 240 | 7·1 | 3·8 | 3·3 | 13 | 101 | Diabetic. |
| 99 | 54 | M. | — | Myelomatosis | | N | — | 2·11 | 5·5 | 154 | 6·5 | 3·9 | 2·6 | 8 | 67 | Bronchopneumonia. |
| 100 | 76 | M. | — | „ | | N | — | 1·83 | 8·6 | 82 | 10·4 | 3·0 | 7·4 | 8 | 123 | — |
| 101 | 50 | M. | — | Hodgkin's disease | | B | — | 1·52 | 9·8 | 98 | 6·1 | 4·3 | 1·8 | 7 | 30 | — |
| 102 | 44 | F. | — | „ | | B | — | 1·91 | 4·0 | 135 | 4·5 | 2·8 | 1·7 | 10 | 7 | — |
| 103 | 65 | M. | — | Myeloid leukaemia | | Cl | — | 1·80 | 15·0 | 214 | 6·0 | 3·5 | 2·5 | 14 | 65 | Urethane. |
| 104 | 40 | F. | — | „ „ | | Cl | — | 1·23 | 12·8 | 55 | 7·0 | 4·4 | 2·6 | 4 | 16 | X-ray therapy. |
| 105 | 70 | M. | — | Aleukaemic leukaemia | | Cl | — | 1·65 | 13·6 | 82 | 6·6 | 3·5 | 3·1 | 4 | — | — |
| (b) *Non-malignant.* | | | | | | | | | | | | | | | | |
| 114 | 19 | M. | | Chronic nephritis | | Cl | — | 1·45 | 9·3 | 132 | 5·8 | 3·3 | 2·5 | 14 | 70 | — |
| 115 | 23 | M. | | Generalized tuberculosis | | Cl | — | 2·56 | 5·8 | 250 | 7·3 | 3·9 | 3·4 | 14 | — | — |
| 116 | 39 | M. | | Actinomycosis | | Cl | — | 2·18 | 5·2 | 239 | 5·8 | 2·9 | 2·9 | 14 | 44 | — |
| 117 | 57 | M. | | *B. coli* infection of wound | | Cl | — | 2·40 | 5·0 | 219 | 6·0 | 2·9 | 3·1 | 12 | 76 | — |
| 118 | 22 | M. | | Rheumatic fever | | Cl | — | 1·86 | 8·7 | 202 | 6·2 | 3·8 | 2·4 | 9 | 85 | — |
| 119 | 45 | M. | | Subacute rheumatism | | Cl | — | 2·04 | 6·2 | 72 | 6·8 | 3·9 | 2·9 | — | 20 | — |
| 120 | 73 | F. | | Early Paget's disease | | Cl, R | — | 1·39 | 10·5 | 72 | 6·2 | 4·0 | 2·2 | 14 | 4 | — |
| 121 | 41 | M. | | Varicose veins | | Cl | — | 1·51 | 6·3 | 85 | 6·7 | 4·4 | 2·3 | — | — | — |
| 122 | 22 | M. | | Anxiety state | | Cl | — | 1·30 | 9·0 | 61 | 6·5 | 4·2 | 2·3 | — | 2 | — |
| 124 | 19 | M. | | Hydatid cyst of liver | | B | — | 1·52 | 8·7 | 60 | 7·6 | 4·7 | 2·9 | 11 | — | — |

(For symbols see p. 385.)

TABLE IX.—*Summary of Results.*

(First analysis on each patient only.)

| Group. | LCCP. + | LCCP. − | IAI. + | IAI. − | P + | P − | E.S.R. + | E.S.R. − |
|---|---|---|---|---|---|---|---|---|
| Infective hepatitis | 2 | 4 | 1 | 5 | 1 | 5 | 0 | 0 |
| Hepatic cirrhosis | 6 | 0 | 2 | 4 | 0 | 6 | 3 | 0 |
| Benign prostate | 9 | 5 | 6 | 8 | 9 | 5 | 3 | 3 |
| Miscellaneous non-malignant | 12 | 9 | 9 | 12 | 12 | 8 | 8 | 5 |
| Carcinomatosis | 11 | 0 | 10 | 1 | 11 | 0 | 2 | 0 |
| Carcinoma of bronchus | 12 | 1 | 10 | 3 | 12 | 1 | 9 | 0 |
| ,, oesophagus | 2 | 1 | 1 | 2 | 3 | 0 | 0 | 0 |
| ,, prostate | 10 | 4 | 5 | 9 | 8 | 6 | 6 | 3 |
| ,, bladder | 5 | 1 | 3 | 3 | 4 | 2 | 2 | 1 |
| ,, stomach | 4 | 1 | 4 | 1 | 4 | 1 | 2 | 2 |
| ,, colon | 4 | 1 | 2 | 3 | 4 | 1 | 2 | 0 |
| ,, rectum | 4 | 1 | 3 | 2 | 3 | 2 | 1 | 2 |
| Jaundice due to malignant growth | 6 | 0 | 1 | 5 | 3 | 3 | 2 | 1 |
| Miscellaneous malignant | 9 | 5 | 6 | 8 | 8 | 6 | 7 | 1 |
| TOTAL NON-MALIGNANT | 29 | 18 | 18 | 29 | 22 | 24 | 14 | 8 |
| ,, MALIGNANT | 67 | 15 | 45 | 37 | 60 | 22 | 33 | 10 |
| % MALIGNANT CORRECT +ve | 82 | — | 55 | — | 73 | — | 77 | — |
| ,, NON MALIGNANT ,, −ve | — | 38 | — | 62 | — | 52 | — | 36 |

(For symbols see p. 385.)

The total protein, albumin and globulin content of sera from malignant and non-malignant cases were not significantly different. There was no obvious correlation between the serum protein concentrations and results of heat coagulation, mucoprotein or E.S.R. tests, but there was a higher proportion of positive IAI tests in sera from all cases with a low total protein content. This is illustrated in Table X.

TABLE X.—*Relationship between Iodoacetate Index and Total Protein Concentration (all cases).*

| Total protein (g./100 ml.). | No. of cases. | % +ve IAI test. |
|---|---|---|
| <5·5 | 8 | 100 |
| 5·5–5·9 | 30 | 70 |
| 6·0–6·4 | 37 | 41 |
| 6·5–6·9 | 28 | 39 |
| 7·0–7·4 | 21 | 38 |
| >7·4 | 5 | 0 |

An increase in the alkaline phosphatase was usually associated with positive "cancer tests," but no single test appears to be directly correlated with this enzyme. The serum alkaline phosphatase was raised in cases of carcinoma of the bronchus (Table IIIa, No. 13, 17, 18, 19, 20 21), stomach (Table IVa, No. 32), colon (Table IVb, No. 34), rectum (Table IVc, No. 38, 39 and 41), prostate (Table VIa), and biliary tract (Table VII).

The serum acid and alkaline phosphatase concentrations of all patients with benign prostatic hypertrophy were normal, but 10 of the 14 cases with carcinoma of the prostate showed a raised acid phosphatase, and 10 a raised alkaline phosphatase. These raised phosphatase levels are useful in indicating the presence of secondary bone deposits, but like the other tests, they are not of much value in detecting an early carcinoma of the prostate.

The E.S.R. was raised in 80 to 90 per cent of cases (malignant and non-malignant) which gave one or more positive results in the three "cancer tests," but it was raised in 3 cases (Table IIIa, No. 15; Table IVb, No. 33; Table VIIIa, No. 104) which gave negative results. Patients with a normal E.S.R., however, did not necessarily give normal results in these tests.

### DISCUSSION.

The results of this investigation show some of the weaknesses of biochemical tests that have been advocated as diagnostic aids in neoplasia. They agree substantially with others which appeared during the present work (Huggins, Miller and Jensen, 1949; Huggins, Cleveland and Jensen, 1950; Jackson, 1950; Pollak and Leonard, 1950; Duboff, 1950; Bodansky and McInnes, 1950; Homberger, Pfeiffer, Page, Rizzoni and Benotti, 1950; Winzler and Smyth, 1948; Kelley, Good and McQuarrie, 1950; Gilligan, Rothwell and Warren, 1950; Finnegan, Brockland, Meuther, Hawk, Inkley and Thoma, 1950). The heat coagulation, iodoacetate and mucoprotein tests, which were studied in this work, are not sufficiently sensitive to detect incipient tumours, are not sufficiently reliable to exclude or to confirm a diagnosis, and generally speaking, their response is not proportional to the severity of the disease. In a variety of diseases they give false positive results. For instance, in the present series false positive results were obtained in bronchitis, cystitis, infective hepatitis, cirrhosis and actinomycosis. Of all the tests, the acid phosphatase activity of the serum appears the most useful in that it helps in the diagnosis of carcinoma of the prostate with metastases.

In various diseases the three tests gave results which conformed to a general pattern, but they differed in their sensitivity towards the change in the serum. Usually they acted similarly, giving either positive or negative results. This behaviour was not invariable, for in some diseases such as cirrhosis, positive heat coagulation tests occurred with normal mucoprotein concentration. It is, therefore, possible that the tests measure different changes which usually occur at the same time or different aspects of one change.

In carcinomatosis, the thermal coagulation (LCCP), the iodoacetate test (IAI) and the mucoprotein test (P) were consistently positive, but their degree of positivity appeared unrelated to the extent of the malignant invasion. For instance, in Case 8 (Table II), of which massive metastases were a feature, LCCP was only slightly abnormal and IAI borderline. There is usually little doubt about the diagnosis of carcinomatosis, but the detection of a single neoplasm is more difficult and urgent. In cases of carcinoma of bronchus (Table IIIa, No. 15), carcinoma of stomach (Table IVa, No. 31), carcinoma of colon (Table IVb, No. 33), carcinoma of rectum (Table IVc, No. 39), carcinoma of prostate (Table VIa, No.48, 50), carcinoma of breast (Table VIIIa, No. 92), epithelioma of lip (Table VIIIa, No. 94) and carcinoma of penis (Table VIIIa, No. 95), all three tests have given completely negative results. This failure of the tests shows up their limitations, and suggests that neither one test nor a combination of them would detect with any certainty neoplasia in an early stage of its growth.

It would seem that serum undergoes changes during an infection somewhat similar to those in malignant disease. For in most of our cases of non-malignant disease infection was a feature, and in the majority of them the three tests gave positive results. The positivity of the tests bore no relation to the severity of the infection, so that it was impossible to judge the contribution infection might be making to any result on a malignant case. In this respect flocculation tests and white cell counts gave no help. It is therefore not surprising that the tests did not differentiate between carcinoma of the bronchus and chronic chest infections. However, it will be observed that whereas the value of the serum alkaline phosphatase was within the normal range in the non-malignant group, it was abnormal in 6 of the 13 cases of carcinoma of bronchus.

No biochemical test has so far been suggested for differentiating benign hyperplasia and malignant disease of the prostate, although the serum acid phosphatase determination is of value in establishing the diagnosis of secondary metastases. In this survey (Table VIa, b) the heat coagulation and iodoacetate tests and the estimation of mucoproteins gave normal and abnormal results in both groups. In view of the effect of infections upon these tests it would be expected that positive results would occur in the non-malignant group, since many of these elderly men had chronic infections as well as enlarged prostates. The serum proteins were of no practical value in differentiating the two conditions, but the serum acid phosphatase was raised in 10 of the 14 cases of carcinoma of the prostate and in none of the cases of benign hyperplasia. The E.S.R. was increased in 6 of the 9 cases of malignant prostate and in 3 of the cases of benign hyperplasia. It would appear that the serum acid phosphatase remains the most useful of the tests used in this survey in establshing the diagnosis of metastases, although it is of little value in the detection of early carcinoma of the prostate.

In the experience of the authors (Higgins and O'Brien, unpublished data) a small proportion of cases of infective hepatitis and cirrhosis give results which do not fit in with the general biochemical picture, and occasional sera from cases of obstructive jaundice show alkaline phosphatase levels which confuse rather than clarify the diagnosis ; such a case is No. 128 (Table VIIa). In these cases weight is given to the flocculation tests, but even these often give borderline results, especially in cases of obstructive jaundice of long duration. It was in the hope of aiding the diagnosis of these cases that the heat coagulation, iodoacetate and mucoprotein tests were done in 6 cases of infective hepatitis, 6 of cirrhosis and 6 of malignant obstruction. The results have not added to the certainty of diagnosis. The heat coagulation test was positive in all cases of malignancy, but was also positive in all 6 cases of cirrhosis and in 2 cases of infective hepatitis. When sera from some cases of cirrhosis were heated the resulting gels were turbid and consisted of two phases—a solid protein " curd " and water. These solutions were similar to those produced by Jensen, Hospelhorn, Tapley and Huggins (1950) on heating albumin in acid solution. These gels were quite different from those obtained from normal sera, and the heat coagulation and iodoacetate tests have, therefore, little meaning with these solutions. The mucoproteins show some unexpected results in that positive results were obtained in one very serious case and one very mild case of hepatitis (Table VIIb, No. 78, 79), negative results in the cirrhosis group, and variable results in the malignant group.

Physical, chemical, enzymatic and immunological investigations have shown the diversity of change in relative proportions and in their nature which plasma proteins undergo in disease. Even more subtle changes may yet be revealed as methods become more delicate and specific. How far these changes represent a general response of the body to disease or one peculiar to one disease, is still to be decided. Of the tests which have been described, the positive response of the iodoacetate test has been attributed to changes in the molecules of protein produced by the body in repsonse to stress of illness (Bodansky and McInnes, 1950). Another suggestion is that the iodoacetate test provides an index of tissue destruction or some form of trauma. These views may be applied to other "cancer tests," and if true would rob them of real value as diagnostic aids in cancer. If some general metabolic change is responsible for positive results in these tests, it is not surprising that abnormal results are found in diseases other than cancer. Nor is it surprising that some sera from cancer patients give normal results, since it is difficult to understand how some isolated neoplasm, such as epithelioma of the lip, could affect protein metabolism.

Of the changes in plasma proteins in disease and of many of the reactions used to reveal them there is still much to be learned. The deficiency in thermal coagulation, as shown by the heat test, may be due to an alteration in the albumin molecule, which is possibly associated with a change in the number of free sulphydryl groups. No gross change in the amino-acid composition of the albumin in these sera has been detected (Huggins, Jensen, Player and Hospelhorn, 1949). Heat denaturation of protein may, however, involve a breaking of hydrogen bonds which hold the protein molecule in its natural form, thus causing the alteration in its configuration and the formation of new hydrogen bonds between carboxyl and amino groups in neighbouring polypeptide chains (Mirsky and Pauling, 1936). Possibly the protein molecule, produced by the body in response to, or because of, the disease, differs from the normal protein in its arrangement of the amino-acid groups, resulting in a different molecular pattern and a differing availability of the carboxyl and amino groups. Jensen, Hospelhorn, Tapley and Huggins (1950) have suggested that iodoacetate reacts with the sulphydryl groups in the albumin molecule, thus introducing a negatively charged carboxyl ion which prevents lateral binding of protein molecules on heating, and that this inhibits coagulation. The decreased amount of iodoacetate necessary to prevent coagulation of sera from diseased patients may be associated with a decreased number of sulphydryl groups. This theory is supported by the report of Schoenbach, Weissman and Armistead (1951) that the sulphydryl content of serum protein from some patients with malignant and other diseases is subnormal. However, as shown in Table XI, a decreased serum protein concentration is usually associated with a positive iodoacetate test. Similar effects were noted by Huggins, Miller and Jensen (1949) and Duboff (1950). It would therefore appear that the reaction is not a simple combination with sulphydryl groups. It is possible that electrostatic forces in the protein-depleted serum are altered so that less iodoacetate is required to bring about a sufficiently negative charge to hinder coagulation. With the elucidation of points such as these, the value of biochemical tests in diagnosis may eventually be properly assessed.

Whatever may be the nature of the change in the protein molecule responsible for the abnormal results of heat coagulation, iodoacetate and mucoprotein tests, it occurs in malignant and non-malignant disease to an extent often disproportionate to the severity of the disease. The tests like the E.S.R. indicate an abnormality of unspecific and as yet undefined nature. Added to this weakness, they often give negative results in cases of neoplasia so that they have little value as "screening tests." Of the other determinations made in this investigation, the serum protein concentration is of little value in the differential diagnosis of malignant disease, and in many cases no guide to the severity of the condition. The acid phosphatase is of value in the diagnosis of carcinoma of the prostate with metastases, and the alkaline phosphatase a useful aid in the diagnosis of liver or bone disease.

In future work it is hoped to investigate more closely the nature of the change in the plasma protein in malignant disease and to correlate it with biochemical studies upon biopsy specimens.

### SUMMARY.

1. The heat coagulation and iodoacetate tests and estimation of mucoproteins have been performed, together with other biochemical tests, upon 25 normal subjects, 82 patients with neoplasia and 47 patients with non-malignant diseases.

2. The heat coagulation test (LCCP) was positive in 82 per cent of carcinoma cases and 62 per cent of non-malignant cases.

3. The iodoacetate test (IAI) was positive in 55 per cent of carcinoma cases and 38 per cent of non-malignant cases.

4. The mucoprotein concentration (P) was increased in 73 per cent of carcinoma cases and 48 per cent of non-malignant cases.

5. No correlation was found between the degree of positivity of these tests and histology or extent of the carcinoma. The iodoacetate test, however, appeared to be related to the serum protein concentration.

6. This survey shows that these tests add little to the laboratory diagnosis of cancer.

The authors' thanks are due to the consultant staff of the United Oxford Hospitals for permission to quote from the notes of their patients, and for details of private cases, to Dr. A. H. T. Robb-Smith for permission to quote from pathological reports, and to members of the staff of this department for valuable advice, help and criticism during this work.

### REFERENCES.

BODANSKY, O., AND McINNES, G. F.—(1950) *Cancer*, **3**, 1.
BRODERS, A. C.—(1925) *Minn. Med.*, **8**, 726.
BROUGHTON, P. M. G., HIGGINS, G., AND O'BRIEN, J. R. P.—(1951) *Biochem. J.*, **49**, xlviii.
DUBOFF, G. S.—(1950) *Cancer Res.*, **10**, 213.
FINNEGAN, J. V., BROCKLAND, I., MUETHER, R. O., HAWK, B. O., INKLEY, J. J., AND THOMA, G. E.—(1950) *J. Lab. clin. Med.*, **35**, 708
GILLIGAN, D, R., ROTHWELL, J. T., AND WARREN, S.—(1950) *New Engl. J. Med.*, **242**, 807.
GUTMAN, A. B.—(1942) *J. Amer. med. Ass.*, **120**, 1112.
*Idem*, AND GUTMAN, E. B.—(1938) *J. clin. Invest.*, **17**, 473.
HERBERT, F. K.—(1946) *Quart. J. Med.*, **15**, 221.
HOMBERGER, F.—(1950) *Cancer*, **3**, 143.
*Idem*, PFEIFFER, P. H., PAGE, O., RIZZONI, G. O., AND BENOTTI, J.—(1950) *Ibid.* **3**, 15.
HOWE, P. E.—(1921) *J. biol. Chem.*, **49**, 109.
HUGGINS, C., CLEVELAND, A. S., AND JENSEN, E. V.—(1950) *J. Amer. med. Ass.*, **143**, 11.
*Idem*, JENSEN, E. V., PLAYER, M. A., AND HOSPELHORN, V. D.—(1949) *Cancer Res.*, **9**, 753.
*Idem*, MILLER, G. M., AND JENSEN, E. V.—(1949) *Ibid.*, **9**, 177.

JACKSON, E.—(1950) *Brit. med. J.*, ii, 1201.
JENSEN, E. V., HOSPELHORN, V. D., TAPLEY, D. F., AND HUGGINS, C.—(1950) *J. biol. Chem.*, **185**, 411.
KELLEY, V. C., GOOD, R. A., AND MCQUARRIE, I.—(1950) *Pediatrics*, **5**, 824.
KING, E. J., AND ARMSTRONG, A. R.—(1934) *Canad. med. Ass. J.*, **31**, 376.
MACLAGAN, N. F.—(1944*a*) *Brit. J. exp. Path.*, **25**, 15.—(1944*b*) *Ibid.*, **25**, 234.
MIRSKY, A. E., AND PAULING, L.—(1936) *Proc. nat. Acad. Sci., Wash.* **22**, 439.
PAPANICOLAOU, G. N., AND TRAUT, H. F.—(1943) 'Diagnosis of Uterine Cancer by the Vaginal Smear.' New York (The Commonwealth Fund).
POLLAK, O. J., AND LEONARD, A.—(1950) *J. Amer. med. Ass.*, **142**, 872.
RAGINS, A. B.—(1934) *J. Lab. clin. Med.*, **20**, 902.
SCHOENBACH, E. B., WEISSMAN, N., AND ARMISTEAD, E. B.—(1951) *J. clin. Invest.*, **30**, 762.
SHEN, S. C., AND HOMBERGER, F.—(1950) *Cancer*, **3**, 36.
WEICHSELBAUM, T. E.—(1946) *Amer. J. clin. Path., Tech.*, **16**, 40.
WESTERGREN, A.—(1921) *Acta med. scand.*, **54**, 247.
WINZLER, R. J., DEVOR, A. W., MEHL, J. W., AND SMYTH, I. M.—(1948) *J. clin. Invest.*, **27**, 609.
*Idem* AND SMYTH, I. M.—(1948) *Ibid.*, **27**, 617.
YOUDEN, W. J.—(1950) *Cancer*, **3**, 32.